# TIAR and FMRP shape pro-survival nascent proteome of leukemia cells in the bone marrow microenvironment

**DOI:** 10.1016/j.isci.2023.106543

**Published:** 2023-03-31

**Authors:** Magdalena Wolczyk, Remigiusz Serwa, Agata Kominek, Agata Klejman, Jacek Milek, Marta Chwałek, Laura Turos-Korgul, Agata Charzyńska, Michal Dabrowski, Magdalena Dziembowska, Tomasz Skorski, Katarzyna Piwocka, Paulina Podszywalow-Bartnicka

**Affiliations:** 1 Laboratory of Cytometry, Nencki Institute of Experimental Biology Polish Academy of Sciences, 02-093 Warsaw, Poland; 2 Proteomics Core Facility, IMol Polish Academy of Sciences, 02-097 Warsaw, Poland; 3 ReMedy International Research Agenda Unit, IMol Polish Academy of Sciences, 02-097 Warsaw, Poland; 4 Animal House, Nencki Institute of Experimental Biology Polish Academy of Sciences, 02-093 Warsaw, Poland; 5 Laboratory of Molecular Basis of Synaptic Plasticity, Centre of New Technologies, University of Warsaw, 02-097 Warsaw, Poland; 6 Laboratory of Bioinformatics, Nencki Institute of Experimental Biology Polish Academy of Sciences, 02-093 Warsaw, Poland; 7 Fels Cancer Institute for Personalized Medicine, Temple University Lewis Katz School of Medicine, Philadelphia, PA 19140, USA

**Keywords:** Biochemistry, Molecular biology, Cancer, Proteomics

## Abstract

Chronic myeloid leukemia (CML) cells circulate between blood and bone marrow niche, representing different microenvironments. We studied the role of the two RNA-binding proteins, T-cell-restricted intracellular antigen (TIAR), and the fragile X mental retardation protein (FMRP) in the regulation of protein translation in CML cells residing in settings mimicking peripheral blood microenvironment (PBM) and bone marrow microenvironment (BMM). The outcomes showed how conditions shaped the translation process through TIAR and FMRP activity, considering its relevance in therapy resistance. The QuaNCAT mass-spectrometric approach revealed that TIAR and FMRP have a discrete modulatory effect on protein synthesis and thus affect distinct aspects of leukemic cells functioning in the hypoxic niche. In the BMM setup, FMRP impacted metabolic adaptation of cells and TIAR substantially supported the resistance of CML cells to translation inhibition by homoharringtonine. Overall, our results demonstrated that targeting post-transcriptional control should be considered when designing anti-leukemia therapeutic solutions.

## Highlights

• Leukemia cells are resistant to treatment in hypoxic bone marrow microenvironment
• FMRP and TIAR modulate nascent proteome in respect to external conditions
• FMRP and TIAR influence synthesis of proteins related to different biological processes
• TIAR is detrimental for the resistance to homoharringtonine in hypoxia

## Graphical abstract

**Figure:**
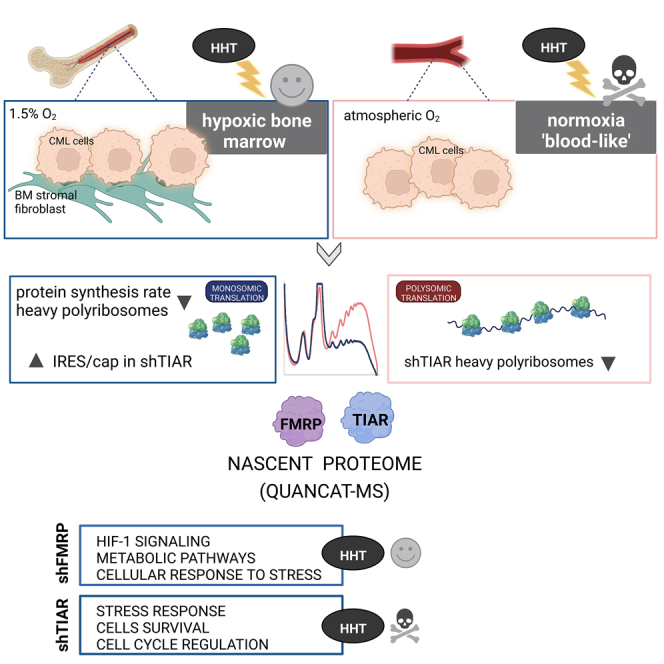

## Introduction

Chronic myeloid leukemia (CML) is driven by the expression of oncogenic kinase BCR-ABL1 in myeloid precursors. The leukemic cells reside in distinct microenvironments: the bone marrow microenvironment (BMM) and peripheral blood microenvironment (PBM). The bone marrow niche is characterized by lower oxygen availability (hypoxia) compared to blood and the presence of stromal cells. Such extracellular context induces leukemia cells' quiescence, which impacts CML cells’ sensitivity to pharmacological treatment.^1^ The main factors that support therapy resistance of leukemic cells are hypoxia,^2^ cytokines secreted by neighboring cells^3^^,^^4^^,^^5^ and direct interaction with stromal cells.^6^^,^^7^^,^^8^ The hypoxia-driven effect could be attributed to the induction of metabolic alterations called the Warburg effect^9^^,^^10^ (reviewed in^11^^,^^12^) as well as proteomic changes.^13^ In this aspect, the translation step might play a key role, based on the discrepancy between the mRNA and protein profile in hypoxic cells.^14^ Despite the available data demonstrating the effect of hypoxia on the translation (reviewed in^15^), it has not been shown how the physiology-relevant interaction of leukemic cells with bone marrow stroma under hypoxic conditions influences the proteome of CML cells. This would shed light on the mechanisms of drug resistance in cancer cells.

Circulation of leukemic blasts between PBM and BMM could induce stress and requires accommodation. The ability to adapt the cellular proteome to the current demands depends on the transcriptional and post-transcriptional regulation. Various factors are instrumental in the steps preceding the protein synthesis, such as the DNA transcription, mRNA splicing, editing, regulation of stability versus degradation, or accessibility to translation,^16^^,^^17^ among which the RNA-binding proteins (RBPs) are key factors (reviewed in^18^). Some RBPs, such as T-cell-restricted intracellular antigen (Tia) proteins (TIAR and TIA-1) and the fragile X mental retardation protein (FMRP)^19^ are activated on the cellular stress response, such as the integrated stress response (ISR). Deregulation of RBPs’ interaction with RNA leads to disorders such as cardiovascular and neurodegenerative diseases, diabetes, and cancer.^18^ The significance of FMRP protein was shown in neuronal cells^20^^,^^21^^,^^22^^,^^23^ and in various cancers.^24^^,^^25^^,^^26^^,^^27^ However, it has never been studied in leukemia. TIA-1 and TIAR are mutated across multiple cancer types and may function as tumor suppressors.^28^^,^^29^ Low expression of Tia correlated with poor prognosis in patients with lung squamous cell carcinoma, whereas its overexpression inhibited proliferation and induced apoptosis in HEK293 cell line.^30^ However, a BCR-ABL1-induced increased level of TIAR supported the oncogene effect in murine CML cells.^31^

TIAR and FMRP regulate different cellular processes depending on the cellular location. The mRNA-bound RBPs shuttle between the nucleus and the cytoplasm.^32^^,^^33^ FMRP and TIAR recognize distinct motives in targeted mRNA – for FMRP it is a G-quadruplex structure,^34^ whereas TIAR binds to AU-rich element (ARE) site^35^ and the 5′-TOP motif (5′terminal oligopyrimidine) in mRNAs encoding ribosomal proteins.^36^ Tia proteins are involved in the alternative splicing of pre-mRNAs in the nucleus^37^^,^^38^^,^^39^ and stress granules formation in the cytoplasm.^40^^,^^41^^,^^42^ In addition, TIAR relocates from the nucleus to the cytoplasm in response to apoptotic cell death^43^ and associates with stress-induced nuclear foci, known as G2/M transition granules, to arrest the cell cycle at the G2/M checkpoint.^44^ Under stress conditions, FMRP locates in so-called P-bodies.^45^^,^^46^ In neurons it regulates synaptic translation of selected mRNAs by polyribosomes stalling^47^^,^^48^ and may inhibit cap-dependent translation.^49^

The influence of BMM on the action of the RBPs involved in the cellular stress response is still an unexplored field of leukemia-therapy resistance. Understanding these changes might be a key to overcoming the resistance of CML cells within the bone marrow niche. Herein, we present how interaction with stromal cells, in the hypoxic microenvironment, affects the profile of synthesized proteins in the leukemic cells, focusing on the role of TIAR and FMRP proteins. Finally, we demonstrate that the activity of TIAR but not FMRP could be supportive in response to treatment with a translation targeting inhibitor like homoharringtonine (HHT). Our results point to the significance of TIAR-dependent modulation of translation in PBM and BMM.

## Results

### Microenvironment impacts signaling in the cells

**Figure 1:**
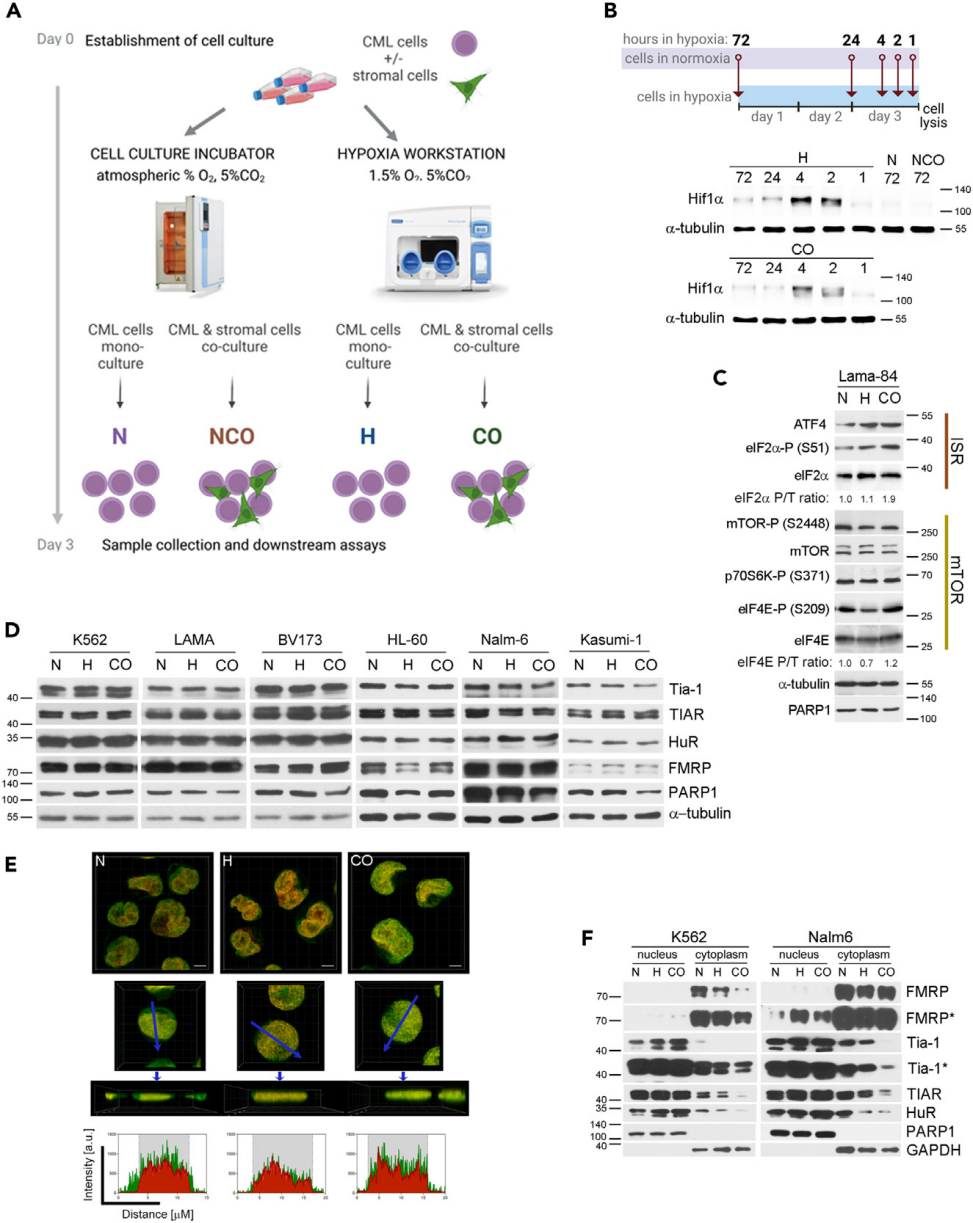
Effect on the cellular signaling related to translation control of *in vitro* experimental setup that reflects blood and bone marrow like conditions The effect of 72 h culture in N, H, CO or NCO checked in human leukemia cell lines K562, Lama-84, BV173, HL-60, Nalm6, Kasumi-1. (A,B upper panel) Scheme of the experiment. (B–D, F) Western blotting analysis in total cellular lysates of (B) hypoxia inducible factor (HIF) at different time points; (C) proteins of the integrated stress response (ISR) and mammalian target of rapamycin (mTOR) pathways; (D) selected RNA binding proteins. (E) Intracellular distribution of HuR in K562 by immunostaining imaged using confocal microscope: Upper panel – a single 0.50 μm stack, HuR (green), DNA (red), white bar = 5 μm; middle panels – 3 dimensional view of overlaid stacks from top and side, blue arrow = place and direction of line profile analysis; bottom panel – line profile analysis of fluorescent signal intensity, gray region marks nucleus. (F) Analysis of nucleus and cytoplasm fractions; ∗ - longer exposition. (B–F) Representative images presented, n = 3.

To study the impact of the hypoxic bone marrow microenvironment (BMM), leukemia cells were co-cultured with human fibroblasts from the bone marrow stroma (CO) in hypoxic conditions (1.5% O_2_ and 5% CO_2_) (Figure 1A), as in our previous studies.^5^^,^^7^^,^^50^ Simultaneously, cells were maintained in mono-culture in standard growth conditions (N) (atmospheric O_2_ and 5% CO_2_) - an established setup broadly used to reflect the physiological blood-like microenvironment (PBM). To distinguish between effects evoked by presence of stromal cells or the hypoxia itself, CML cells were also cultured in hypoxic mono-culture (H). The main focus of our work is on hypoxic BMM, because it encompasses factors that play critical role in therapy resistance of CML in bone marrow — hypoxia and contact with stromal cells. However, in some experiments we also employed experimental setup of co-culture under normoxia (NCO) to follow impact of stromal cells without hypoxia.

It has been well documented that response to hypoxia implies increased level of hypoxia inducible factor (HIF) 1α in cells. Decrease in oxygen availability causes stabilization of HIF1α protein, that is no longer directed for degradation,^51^ and acts as transcription factor regulating expression of genes essential for adaptation to hypoxia.^52^ Moreover, it has been reported that HIF1α translation as well as transcription is downregulated on cellular stress response^53^ and eIF2α phosphorylation.^54^^,^^55^ We first compared short versus long term effect of 1.5% O_2_ by checking induction of HIF1α after different times of incubation (experiment design explained in Figure 1B upper panel). The cells were seeded in all 4 setups on the same day in mono- or co-culture. For 72 h adaptation period the cells were transferred to hypoxia immediately. After 48 h of growth in normoxia the cells were transferred to hypoxia to check impact of 24 h incubation. On day 3, before lysis, the cells that had been in normoxia for 68, 70 and 71 h were placed in hypoxic conditions for 4, 2 and 1 h, respectively. This way, we compared impact of hypoxia versus normoxia in cells that were seeded at the same day and grew the same period of time after handling to avoid the impact of factors such as cells centrifugation or density on HIF1α induction in case of short time incubation. The results showed that in hypoxic conditions HIF1α protein level is rapidly elevated but the effect seems transient, as it is decreasing with longer time of incubation in mono-as well as co-cultured cells (Figure 1B). To avoid the effect of short-term responses and study cells well-adapted to the conditions (resembling physiology), cells had been grown in given experimental setup for 72 h before analysis. In K562 cells under hypoxia detection by annexin A5, a hallmark of apoptosis process, increased from 4.6 ± 0.9% in N to 9.1 ± 2.6% in H (p = 0.045), but was not changed in CO at 4.8 ± 1.3% (p = 0.065) n = 4 (Figure S1A). In agreement with observations from normoxia^56^^,^^57^ this showed, that presence of stromal cells plays a supportive role for CML cells under hypoxia. In CML cell lines, K562 and Lama-84, hypoxia co-culture increased the number of cells in G0/G1 phase of cell cycle by 7.8 ± 1.9% but the change was not significant (p = 0.176, n = 3). The % of cells in the subG0/G1 increased from 2.1 ± 0.5% to 3.9 ± 1.4% in H (p = 0.106) and 4.7 ± 1.6% (p = 0.062) in CO; n = 4 (Figure S1B). Hypoxic conditions significantly prolonged the population doubling time (Figure S1C) of K562 cells comparing to normoxia in H by 3.9 ± 0.5 h, p = 3.4E-10 and in CO by 7.1 ± 0.8 h, p = 3.1E-11. This could be a result of slower cell cycle progression or a process of cell death that is not detected using annexin A5. Worth noticing, unlike in hypoxia, the interaction with stromal cells in normoxia had no significant impact on the population doubling time of K562 cells (Figure S1D). Taken together, under hypoxic conditions leukemia cells express HIF1α and display reduced population doubling, what implicates that functioning of cells is different in these conditions. For this reason, in most subsequent experiments, to check for significance of stromal cells interaction under hypoxia, a mono-culture in hypoxia is used for reference rather than co-culture in normoxia, where HIF1α is not increased in 72 h adapted cells and population doubling time is not affected (Figure 1B). Altogether, the results suggested changes in the cellular signaling that implicate modification of synthesis of regulatory proteins.^58^

Hypoxia affects mRNA-translation regulatory networks,^15^ based on the mammalian target of rapamycin (mTOR) and integrated stress response (ISR) that lead to phosphorylation of eukaryotic initiation factor (eIF).^59^ Previously, we observed that mild ISR activity in CML cells supported resistance to imatinib^60^ and changed the secretory proteome.^61^ Besides, processes like proliferation and apoptosis have impact on translation and there is a dispute regarding the impact of eIF2α phosphorylation on global translation during cell cycle (reviewed in^62^^,^^63^). Thus, we monitored the activity of ISR and mTOR pathways in our experimental setup. We observed increased phosphorylation of eIF2α subunit at Ser51 residue (S51-P), accompanied by increased level of activating transcription factor 4 (ATF4) on CO (Figure 1C), but no critical effect on the mTOR pathway (Figures 1C and S2A). Taken together, interaction with stromal cells can be accompanied by induction of ISR in leukemia cells. This could decrease HIF1α protein level in cells on long-term incubation in hypoxic conditions (Figure 1B), based on described impact of stress induction on HIF1α translation and transcription.^53^^,^^54^^,^^55^ On the other hand, ATF4 has recently been shown to enhance transcription of HIF1α in long-term hypoxia in pancreatic cancer,^64^ what would explain detection of HIF1α protein in our hypoxia adapted cells (Figure 1B).

### The bone-marrow mimicking conditions effect on the nucleus-cytoplasm shuttling of RBPs

We followed the effect of the microenvironment on the RNA binding proteins (RBPs), which orchestrate the post-transcriptional control (reviewed in^18^^,^^65^^,^^66^^,^^67^^,^^68^). We focused on the ARE-site binding proteins that we have previously studied^31^ and FMRP that recognizes distinct RNA motif but its activity is changed on stress response. The conditions exerted no significant impact on the total cellular level of RBPs tested in human (Figure 1D) and murine (Figure S2B) leukemia model cell lines. However, in human (Figures 1E and 1F) and murine (Figure S2C) cells, the CO setup promoted decrease of the tested RBPs level in the cytoplasm, accompanied by their accumulation in the nucleus (Figures 1E and 1F). This implicates that functionality of the RBPs is different in the BMM because it depends on the cellular compartment^40^^,^^42^^,^^69^ and is modified during cell cycle progression.^44^^,^^70^

### Environmental conditions have an impact on the interaction of TIAR and FMRP with ribosomal proteins

**Figure 2:**
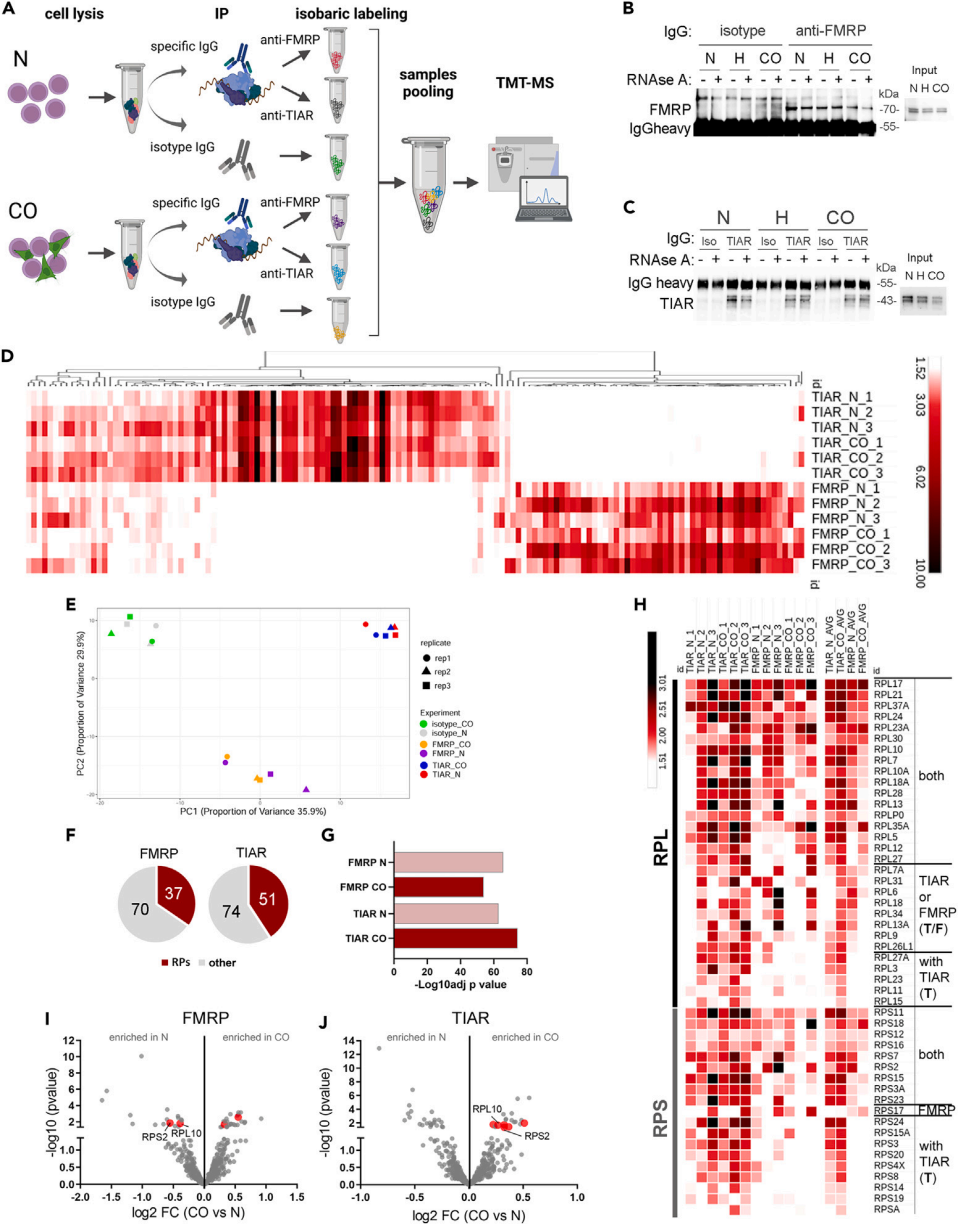
Microenvironment-induced changes in the profile of proteins present in cytoplasmic complexes of TIAR and FMRP proteins Mass spectrometry analysis combined with tandem mass tagging (TMT-MS) was used to study the proteome of complexes immunoprecipitated (IP) from cytoplasm of K562 cells cultured in N or CO setup for 72 h. Profile of proteins isolated by IP with antibody of the same isotype (iso IgG) as the antibody used for IP of TIAR and FMRP (specific IgG) used as a background control. In total 6 isolations from each condition and IP were combined for isobaric labeling and analyzed by mass spectrometry in a single run. (A) Scheme of experiment. (B and C) Western blotting analysis of IP prepared with IgG isotype (iso) and FMRP (B) or TIAR (C) from K562 untreated (−) or treated (+) with RNase A for 1 h; input - signal in the cytoplasmic fraction used for IP. Representative images presented, n = 3. (D and E) Analysis of proteins identified in TMT-MS. Comparison of proteins with average ratio RBP/ISO ≥2 from 3 experiments (1–3 following the sample name) presented as (D) heatmap; legend on the right – in white ratio ≤1.5; in dark red ratio =10; (E) principal component analysis. (F–H) Analysis of proteins with significant (p<0.05) change of ratio RBP/ISO ≥1.5. (F) Number of ribosomal proteins (RP) identified in the samples (both N and CO). (G) Assessment of enrichment in the samples in proteins annotated KEGG:Ribosome03010. (H) Comparison of presence of small (RPS) and large (RPL) ribosomal subunits proteins in the IP complexes of TIAR and FMRP from N and CO conditions – fold change (FC) values of RBP/ISO are presented on the heatmap, n = 3; 1–3 – single experiments, AVG - mean value of n = 3; legend in left up corner – in white FC ≤1.5, in dark red the highest FC >2.5; both - proteins present in complex with TIAR (T) and FMRP (F). (I and J) Impact of CO versus N on RP level in FMRP (I) or TIAR (J) IP complexes; selected RPs in red.

We have specifically focused on TIAR and FMRP proteins, known to be relevant to the protein synthesis process^40^^,^^48^ but recognize different motives in mRNA. Thus, we checked how hypoxic BMM versus PBM conditions influence the proteomic composition of complexes formed by TIAR and FMRP in cytoplasm. To this end, lysis of cells was performed following the protocol designed to isolate protein-RNA complexes for the polyribosome profiling. Using co-immunoprecipitation (IP), mass spectrometry and tandem mass tagging (TMT-MS) (scheme in Figure 2A) we identified and quantified the differences in the presence of proteins in the TIAR and FMRP IP, normalized to control IP with antibody of the same isotype (isoIgG). Similarly to initial observations (Figure 1F), the level of proteins in CO conditions was reduced in the IP analysis with anti-FMRP (Figure 2B) and anti-TIAR (Figure 2C) antibodies. The aside observation is that presence of RNA could impact the proteins self-aggregation in N conditions, but in CO conditions only in the case of FMRP (Figures 2B and 2C).

The protein composition of cytoplasmic complexes pointed to distinct interaction partners of FMRP and TIAR (Figure 2D), however the effect of microenvironment was not significant (Figures 2D and 2E). Analysis of the enrichment in proteins representing functional groups revealed that ribosomal proteins (RPs) in TIAR and FMRP complexes constituted 51 and 37 proteins detected in either condition with a significant signal ratio to isotype control (higher than 1.5), respectively (Figure 2F). The enrichment value of RPs was at a similar level in N conditions, whereas in CO the representation of RPs decreased in the FMRP and increased in the TIAR complexes (Figure 2G). Especially, selected ribosomal proteins (like L17, L21, L37A, L28) interacted independently of environmental conditions with both, TIAR and FMRP (Figure 2H). Several RPs of the large and small subunit interacted preferentially with TIAR but not FMRP (e.g. S3, L11). Noteworthy, a group of RPs interacted with TIAR in CO and with FMRP in N conditions (e.g. L31, L6, L18).

Next, we quantified the effect of bone marrow versus blood-mimicking conditions on the level of proteins in the IP complexes (Figures 2I, 2J and S3A), normalized to the control IP with antibody of the same isotype (isoIgG). Because some ribosomal proteins were detected also in the IP with isotype IgG, the quantification aimed to search for enrichment of ribosomal proteins. This showed that RPS2 and RPL10 interacted preferentially with FMRP in N but not in CO conditions, and with TIAR in CO but not in N conditions (Figures 2I and 2J). Immunoblotting confirmed enhanced level of RPL10 and RPS2 proteins in TIAR (Figure S3B) and FMRP (Figure S3C) IP complexes. Altogether, the obtained data suggested that microenvironment could influence interaction of RBPs with the particular RPs.

### The bone-marrow mimicking conditions limit formation of polyribosomes

**Figure 3:**
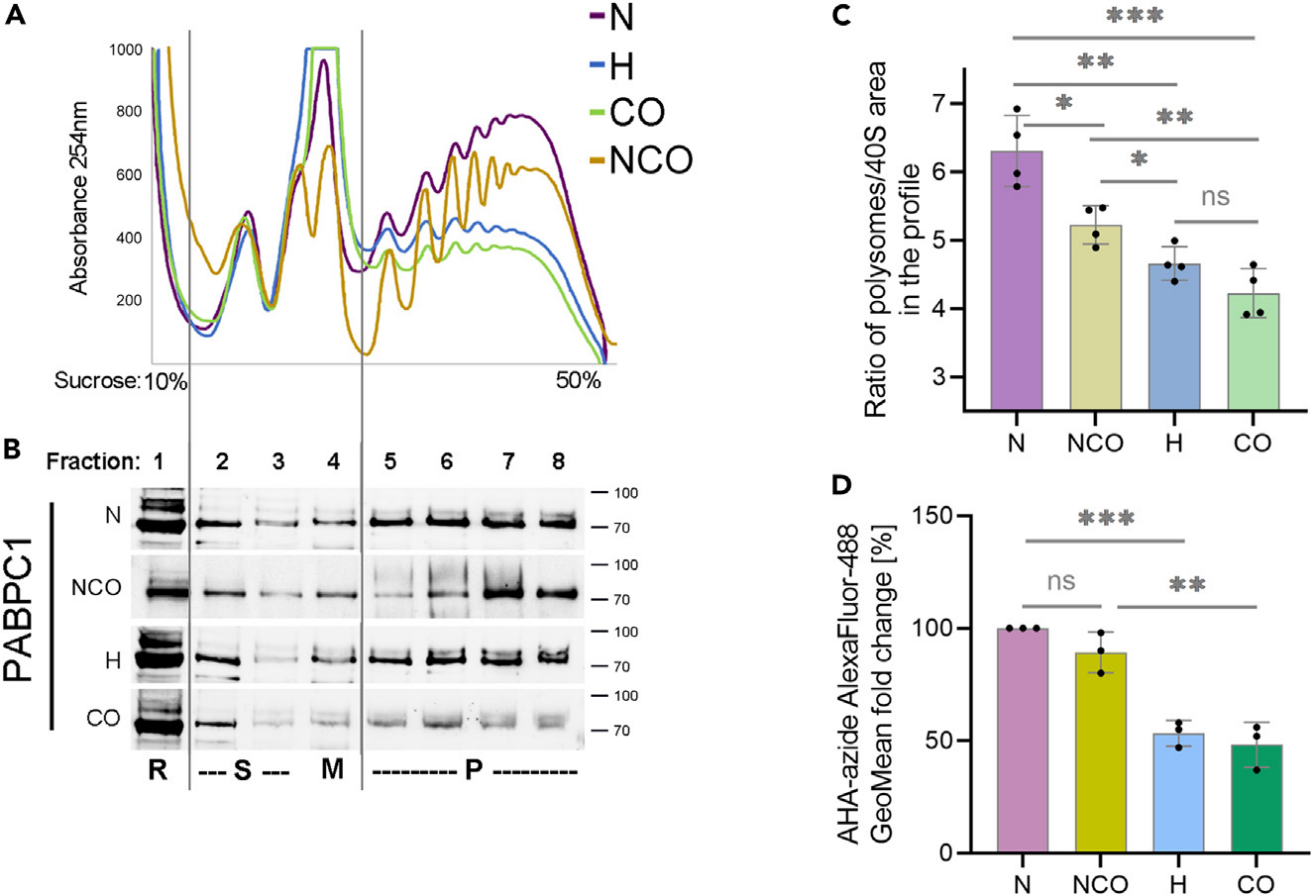
Microenvironment effect on the polyribosome profile K562 cells were cultured in N, H, CO or NCO setup for 72 h. (A) Profile of absorbance monitored at λ = 254 nm during fractionation of sucrose gradient (from top 10% to bottom 50%). Representative image presented, n = 3. (B) The same volume of fractions used for analysis of PABPC1 protein by Western blotting in the fractions. Fractions denoted: R – RNA binding proteins complexes; S – small ribosomal subunit; M− large ribosomal subunit and monosome; P – polyribosomes. (C) Area under polyribosome fractions and small ribosome subunit peak (S, fr. 2) measured using ImageJ and presented as ratio; n = 4. (D) Level of AHA-AlexaFluor 488 labeled proteins in K562 cells from N, H, CO or NCO conditions measured by flow cytometry; n = 3. (C and D) Mean values from at least 3 experiments ± ME are presented; Student’s *t*test to compare cells as indicated by line above bars; ∗p<0.05, ∗∗p<0.005, ∗∗∗p<0.001, ns – not significant.

**Figure 4:**
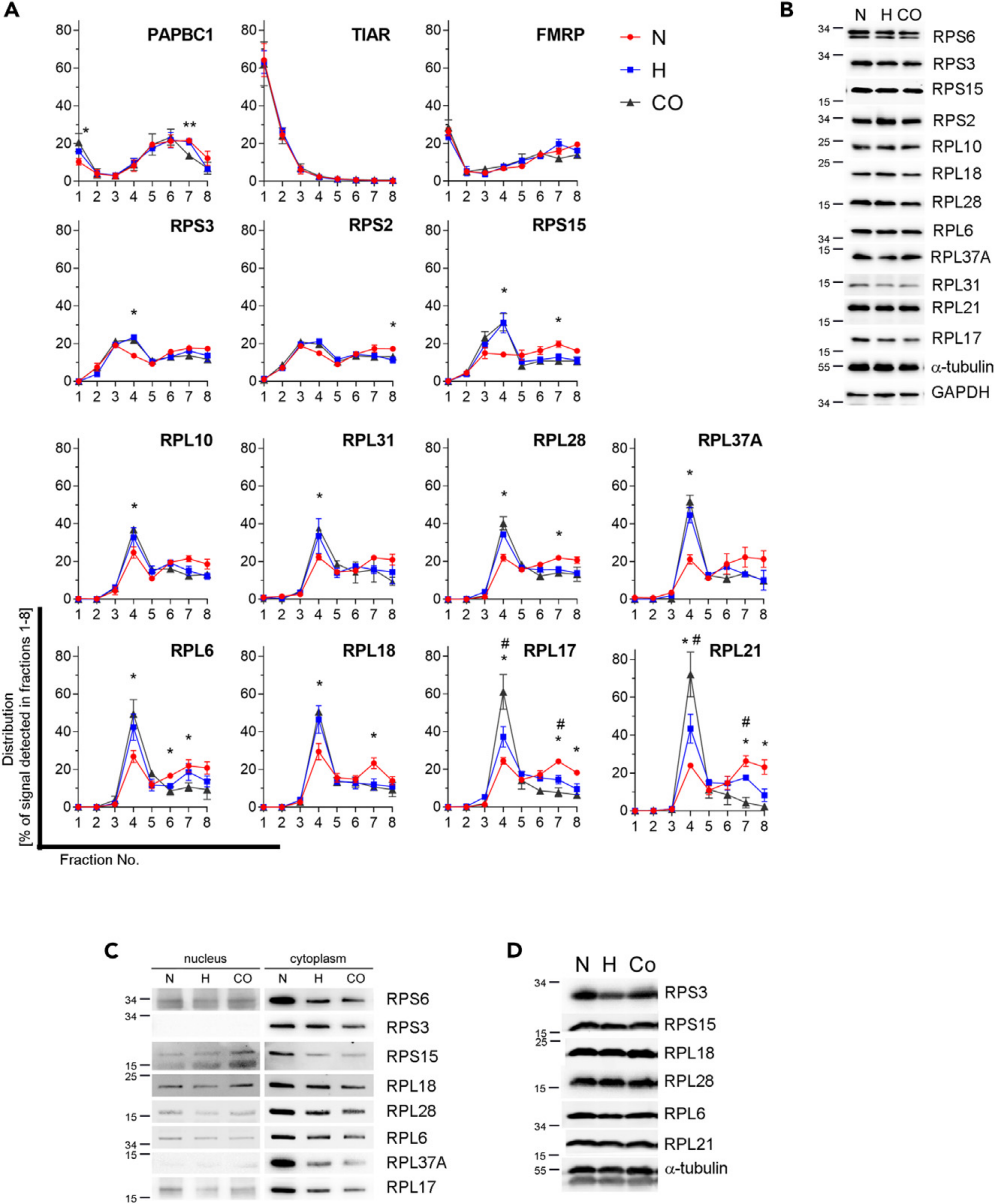
Distribution of ribosomal proteins in polyribosome profile K562 cells were cultured in N, H or CO setup for 72 h before fractionation of polyribosomes in a sucrose gradient. Fractions collected from top 10% to bottom 50% sucrose, numbered 1–8, were analyzed by Western blotting – the same volume of each fraction loaded. (A) Distribution of proteins in the fractions - densitometry of WB results by ImageJ presented as a % of a sum signal intensity of bands in all 8 fractions on a blot, mean values ± ME, n = 3; Student’s *t*test to compare cells in N with H (#) or N with CO (∗); ∗p<0.05, ∗∗p<0.005. (B) Level of RPs in total cell lysates loaded on the gradient. (C) Nucleus and cytoplasm fractions and (D) total cell lysates.

Changes in the activity of signaling pathways (Figures 1B and 1C) suggested modification of translation process on the BMM-mimicking conditions. We checked the impact of leukemia-stroma interaction on the assembly of ribosomes (monosomes) and formation of multi-ribosomal complexes (polyribosomes) on mRNA. According to profiles normalized to the protein input (Figures 4B and S4), hypoxic BMM in comparison to PBM reduced formation of polyribosomes and induced accumulation of monosomes (Figure 3A). Differences in the polysome profile were reflected by changes in the distribution of mRNA poly-A binding protein C1 (PABPC1) in the fractions (Figure 3B) as well as ribosomal proteins (Figure 4). Based on the absorbance profile (Figure 3A) and presence of 28S and 18S rRNA (Figure S5A), the fractions (fr.) were assigned with: R- RNA bingeing proteins complexes (fr. 1), S- mostly small ribosomal subunit (fr. 2–3) or M− monosome (fr. 4) and P – polyribosomes (fr. 5–8). Our results indicate that hypoxic conditions strongly affect polyribosome assembly, what was observed in other cancer cells as well.^14^^,^^71^ Interaction with stromal cells versus mono-culture seemed to reduce formation of polyribosomes in normoxia as well as hypoxia (Figure 3A). To estimate differences, we measured area under peaks of polyribosomes (P) and the small ribosome subunit (S, fr. 2), and calculated its ratio (Figure 3C). The hypoxic BMM versus PBM (CO versus N) conditions reduced polyribosome assembly in CML cells by about 32% (ratio changed by 2.1 ± 0.3, p = 0.0006, n = 4). In case of cells cultured in mono-culture, hypoxia decreased the ratio by ∼26% (1.6 ± 0.3 difference to N, p = 0.0013, n = 4). Interaction with stromal cells comparing to mono-culture displayed weaker impact, significantly reduced formation of polyribosomes in normoxia by ∼16% but not in hypoxia (ratio lowered in N by 1.1 ± 0.3, p = 0.0106, whereas in H by 0.4 ± 0.2, p = 0.0934; n = 4).

To assay relevance of variations in the polyribosome assembly we measured the incorporation of L-azidohomoalanine (L-AHA) to the peptides by using click chemistry and flow cytometry (Figure 3D). Rate of proteins synthesis was reduced by 46.7 ± 3.3%, p = 0.0001, n = 3 in hypoxia versus normoxia mono-culture. Under hypoxia, there was no significant difference between mono- and co-culture, what reflected results from polyribosome profile analysis (Figure 3C). Thus in hypoxic BMM versus PBM translation process was inhibited because of hypoxia-driven impact. Noteworthy, comparison of N to NCO demonstrated that co-culture in normoxia had no significant impact on translation rate in K562 cells (difference by 10.7 ± 5.3%, p = 0.1099, n = 3), despite the effect on the polyribosome assembly (Figures 3A–3C). Possibly, reduction of polyribosomes formation observed in NCO (Figures 3A and 3B) was not sufficient to decrease overall translation efficacy. This might result from a fact that although polyribosomes are major site of efficient translation process,^72^ also monosomes have been demonstrated to actively translate mRNA.^73^^,^^74^ Altogether, performance of translation process together with unaffected population doubling time (Figure S1D) and lack of stabilization of HIF1α protein in long-term cultured cells (Figure 1B) point to considerably different biology of leukemic cells in normoxic versus hypoxic co-culture.

Quantification of proteins distribution in the polysome gradient fractions (Figures 4A, 4B and S5B) showed that FMRP and PABPC1 proteins were detected in all fractions, but mainly in the R and P fractions. On the contrary, TIAR distributed to the R and S fractions. Distribution of TIAR or FMRP was not significantly affected by hypoxia and presence of stromal cells had no additional effect.

As we had identified differences in RPs composition in the complexes of TIAR and FRMP proteins (Figure 2), and had been considering that such complexing could affect the RPs assembly with ribosomes,^41^ we checked the levels of selected ribosomal proteins (identified by TMT-MS) in the polysomal fractions (Figures 4A and 4B; original blots in Figure S5B). There were notable differences in RPs assembly with ribosomes existing as monosomes versus associated in polyribosomes. In the BMM conditions, % distribution of most of the ribosomal proteins to the monosome fraction increased. Under hypoxia, this effect was significantly enhanced by interaction with stromal cells in case of RPL17 and RPL21 that were detected at high level in TIAR and FMRP complexes (Figure 2F). On the contrary, distribution of RPS3, RPL10, RPL31 and RPL37A to the last P fraction of so called ‘heavy’ polyribosomes (fractions 7 and 8) was not significantly affected by either condition. A common feature of the RPs (S3,L10, L31, L37A) is that in hypoxic BMM these were detected at higher level in complex with TIAR than FMRP (Figures 2F–2I). Comparing to N, some of RPs were more in the nucleus in CO than in H (Figure 4C), whilst the total levels were unaffected (Figure 4D).

Altogether, in the hypoxic BMM-mimicking setup comparing to PBM, polyribosome assembly was reduced and accompanied by decreased translation efficiency. Moreover, ribosomal proteins displayed heterogenic response to microenvironment signals.

### Knockdown of TIAR or FMRP exerts effect on the formation of polyribosomes

**Figure 5:**
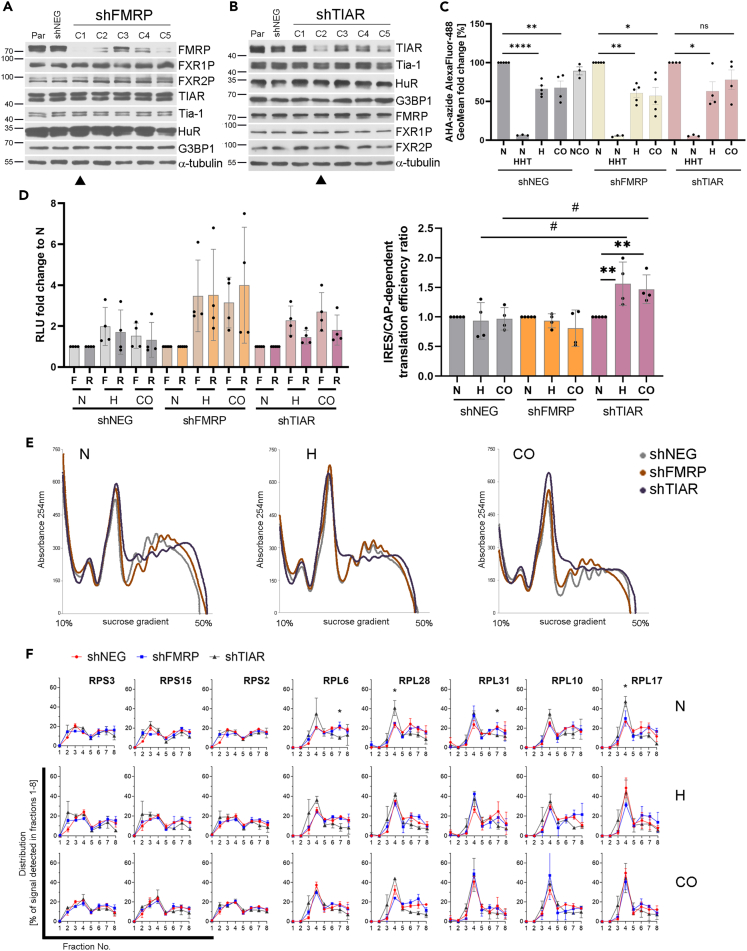
Knock-down of FMRP or TIAR have impact on the translation process (A and B) Total cellular lysates of K562 cells transduced with 5 different lentiviral particles bearing different shRNA (C1-C5) were checked for TIAR (A) and FMRP (B) protein knock-down by Western blotting and compared with parental (Par) and transduced with control virus (shNEG) cells; a black triangle - clones used further. (C) Level of AHA-AlexaFluor 488 labeled proteins in K562 cells from N, H or CO conditions measured by flow cytometry; 100 nM homoharringtonine (HHT) in N for control. (D) IRES- (F) and cap- (R) dependent translation (left panel) and ratio IRES/cap (right panel) tested by dual-luciferase assay. (C and D) Fold change to N conditions, mean values ± ME, n = 4. Student’s *t*test to compare cells in N with H or N with CO (∗) and one-way ANOVA to compare shTIAR to shNEG and shFMRP in H or CO (#); ∗p<0.05, ∗∗p<0.005, ∗∗∗∗p<0.0005, ns – not significant. (E) Profile of ribosomal mono-/polyribosomes separated in sucrose linear gradient 10–50%; absorbance monitored at λ = 254 nm, normalized to protein level in the input. (F) Distribution of ribosomal proteins in the fractions (numbered 1–8) analyzed by Western blotting - densitometry of results by ImageJ as a % of a sum signal intensity of bands in all 8 fractions on a blot; mean values ± ME, n = 3; Student’s *t* test to compare cells shNEG to shTIAR; ∗p<0.05.

A number of reports demonstrated that FMRP can be found in polyribosomes fraction, what we also confirmed (Figures 4A and S5B), and induces ribosomes stalling.^47^^,^^75^ There are, however, no data regarding the effect of TIAR silencing on the translation profile. Besides, impact of the RBPs has never been studied in the context of hypoxic bone marrow conditions. Thus, we stably silenced TIAR or FMRP (selecting the most efficient shRNA from 5 different shRNAs marked as C1-C5 Figures 5A and 5B). Further experiments were performed using cells transduced with shFMRP_C1 or shTIAR_C5 (Figures 5A and 5B). We verified that amount of known interacting partners of knocked-down proteins was unaffected in the selected clones (Figures 5A and 5B). Following data presented by others,^30^^,^^44^ we checked that the knock-down of TIAR or FMRP in K562 cells had no impact on the apoptosis level (Figure S6A), cell cycle progression (Figure S6B) nor population doubling time (Figure S6C) of cells under N, H and CO conditions. As the so far results pointed to altered translation efficiency under hypoxic BMM, we first estimated the level of newly synthesized proteins by measuring the incorporation of L-AHA and click reaction (Figure 5C). In accordance with other presented results, the rate of translation process compared to normoxia was reduced in cells with control transduction (shNEG) by 34 ± 4%, p<0.0001 under hypoxic conditions and not additionally changed on interaction with stromal cells (32.5 ± 8%, p = 0.0041). Knock-down of FMRP or TIAR had no impact on this effect (ANOVA one way p = 0.8636 in H and p= 0.4297 in CO). Considering the decreased population doubling time in H and CO versus N (Figure S1C) and relevance of selective translation in the regulation of cell cycle progression,^62^^,^^63^ we tested whether this reduced efficacy of protein synthesis under hypoxia addressed both, the internal ribosome entry site (IRES) and cap-dependent translation.^76^^,^^77^ In cells transduced with shNEG or shFMRP, the ratio of IRES/cap-dependent translation was not significantly affected by hypoxia, comparing to N setup (Figure 5D). On the contrary, downregulation of TIAR increased this ratio by about 50% under hypoxic conditions (change by 0.56 ± 0.17 p = 0.01, ANOVA one-way p = 0.018 in H and by 0.47 ± 0.11 p = 0.0031, ANOVA one-way p = 0.012 in CO).

Next, we verified whether silencing of FMRP or TIAR modified the polyribosome profile. We superimposed profiles that were normalized to the total amount of proteins in the cellular lysate loaded on the gradient. Silencing of TIAR affected formation of polyribosomes in N and H conditions (Figure 5E, inputs in Figure S7). In hypoxia mono-culture knock-down of FMRP displayed no impact, but in co-culture enhanced formation of polysomes. We checked if changes in the polysome profile are accompanied by different distribution of RPs within the gradient (Figure 5F). Silencing of FMRP had no significant impact on the % distribution of RPs in the fractions. In case of shTIAR, some significant changes were in normoxia. The % content of RPL28 and RPL17 was increased in the fraction of monosome. These proteins were detected at relatively high level in complex with both, TIAR and FMRP. Apart from that, silencing of TIAR reduced the % content of RPL6 and RPL31 in the fraction of heavy polysomes; however, these RPs under N interacted preferentially with FMRP. The data suggest that differences regarding protein distribution arise from affected polysome formation rather than specific RBP-RPs interaction (Figure 2F).

To sum up, knockdown of TIAR or FMRP impacted formation of polyribosomes what could have an influence on the assembly of selected ribosomal proteins to ribosomes associated in mono-versus polyribosomes. Furthermore, silencing of TIAR, but not FMRP, changed the selectivity of translation initiation in hypoxic conditions.

### Change of microenvironment modifies the profile of newly synthesized proteins

**Figure 6:**
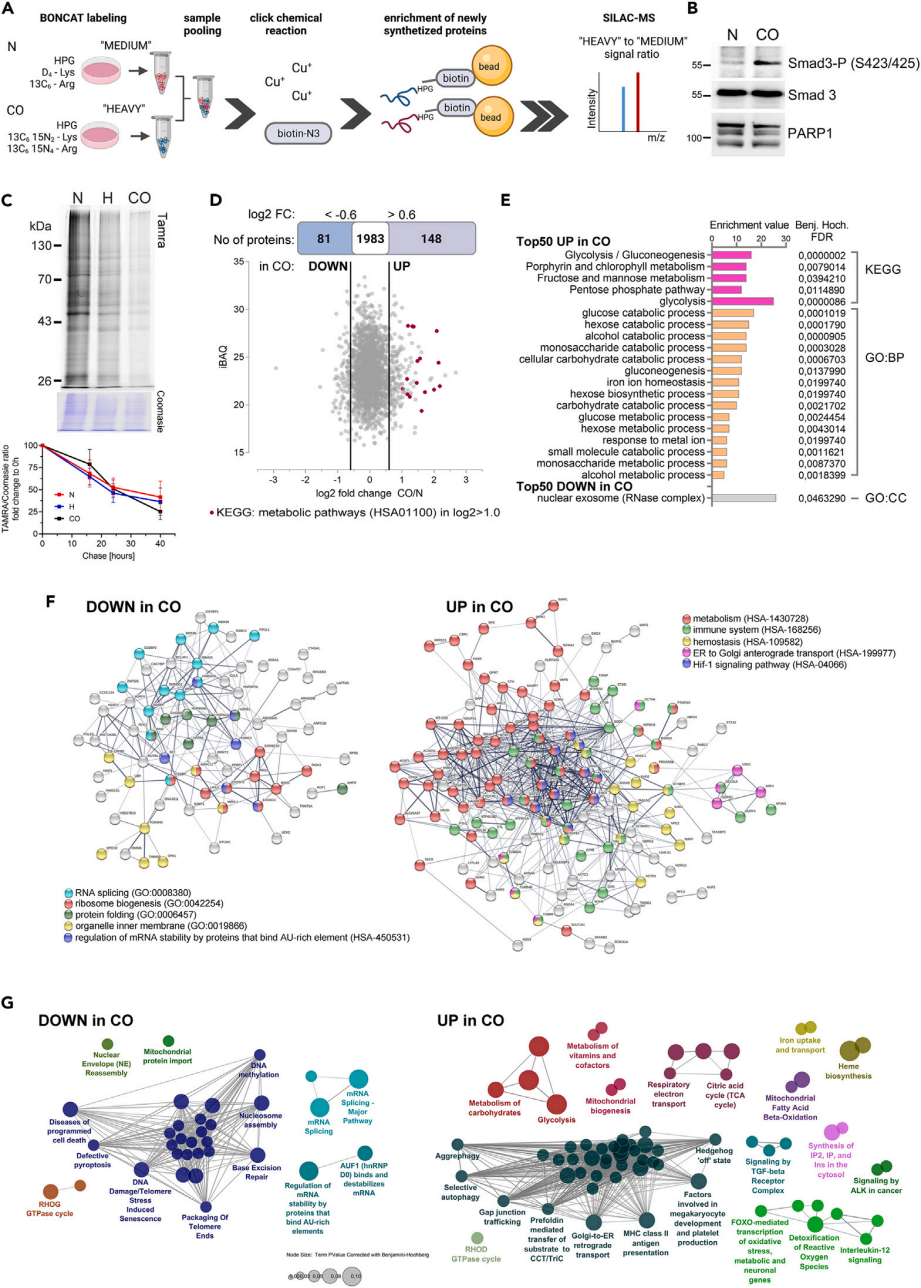
Impact of the bone marrow mimicking conditions on the profile of the synthesized proteins (A) Scheme of the workflow. K562 cells cultured for 72 h in normoxia mono-culture (N) or hypoxia co-culture (CO), followed by incorporation into the synthesized peptides L-homopropargylglycine (L-HPG) and ‘heavy’/’medium’ Lys or Arg for 4 h. Click chemical reaction used to covalently bind biotin from azide to HPG, then nascent peptides enriched by binding to beads. Identification and quantification of the peptides using SILAC-MS based on the ratio of ‘heavy’ to ‘medium’ peptides intensity detected. Results from 3 independent experiments. (B) Protein level in total cellular extract analyzed by Western blotting. (C) Upper panel: Visualization of HPG-enriched proteins labeled in reaction with TAMRA-azide (Cy3 fluorescence channel) separated by SDS-PAGE and stained by Coomassie (bright field). bottom panel: signal intensity after HPG removal at time points 16 h, 24 h and 40 h expressed as % of signal at 0 h; mean values ± ME, n = 3. (D) Comparison of level of proteins according to the intensity Based Absolute Quantification (iBAQ) referred to the intensity fold change in CO versus N expressed as log2 value; black lines at log2+/−0.6 values. Upper graph - number of proteins unchanged (center white box), significantly upregulated (log2>0.6, UP, on the right) or downregulated (log2<−0.6, DOWN, on the left) in CO versus N. Proteins with log2>1.0 assigned to the highly represented KEGG category are marked in pink. (E) Gene Ontology Biological Processes (GO:BP), Cellular Component (GO:CC) and KEGG terms annotated in Perseus with Fisher’s exact test (for 50 proteins ‘Top50’ UP and DOWN with highest log2 change CO versus N); enrichment values with p≤0.05 with a Benjamini-Hochberg (Benj.Hoch.) FDR ≤0.05. (F and G) Protein functional enrichment by (F) string.db - proteins marked according to the legend below the graph; and (G) ClueGO – REACTOME Pathways. Only terms over-represented with Benj.Hoch. FDR ≤0.05.

Our results demonstrated that the hypoxic BMM reduced the general protein synthesis rate. We hypothesize that the BMM-mediated support of therapy resistance relies on the changes in proteome of leukemia cells. Such effect of the bone marrow stroma has never been investigated. Thus we aimed to study physiologically relevant effects exerted by PBM and BMM and shed more light on the cellular functionalities that might play a role in the therapeutic context. Thus, we profiled nascent peptides synthesized in cells in the N and CO setup (Figure 6A) and confirmed activation of TGFβ/Smad3 signaling in K562 in CO (Figure 6B), that supports chemo-resistance.^5^ We visualized relative differences in the nascent proteome metabolically labeled with a bio-orthogonally tagged methionine analogue, L-homopropargylglycine (HPG) under N and CO conditions. We found a noticeable difference in the nascent proteome of cells cultivated in the N, H and CO conditions, visualized by SDS-PAGE using TAMRA-labeled azide in the chemoselective click reaction (Figure 6C). The level of newly synthesized peptides was visibly decreased, whereas the general peptide-specific Coomassie stain was at the similar level. Such reduced degradation was also observed in fish exposed to hypoxia.^78^ Considering that changes in the signaling activity, like mTORC1, could impact both synthesis and degradation rate of proteins,^79^ we additionally verified, that the reduced rate of translation in hypoxic BMM versus PBM was not accompanied by general slower degradation rate/increased protein stability (Figure 6C, bottom graph).

To quantify the magnitude of the decreased translation rate on individual proteins, we next used mass spectrometry analysis of quantitative non-canonical amino acid tagging (QuaNCAT), that combines the bio-orthogonal non-canonical amino acid tagging (BONCAT) with HPG and stable-isotope labeling of amino acids in cell culture (SILAC).^80^ Initially, we tested 1 h and 4 h incubation with HPG in hypoxia mono-culture and observed that longer time increased the number of identified proteins by nearly 10-fold (data not shown). Following data normalization to account for the global reduction in nascent protein levels under CO conditions, amongst 2217 protein groups identified in the experiment, we found 229 with differentially regulated nascent levels. Of those, 81 were synthesized more readily in normoxia, and 148 in the BMM mimicking setup (Figure 6D and the protein list in Table S1). As the lower translation rate was indeed accompanied by changed profile of readily synthesized proteins, we analyzed GeneOntology Biological Processes (GO:BP) and Kyoto Encyclopedia of Genes and Genomes (KEGG) annotations enrichment in respect to all detected proteins with Fisher’s exact test (Figure 6E). Within the group of 50 protein groups with the most pronounced increase (‘Top50 UP’) in nascent levels in CO versus N, several corresponded to enzymes substantial in carbon metabolic processes (marked in pink in Figure 6D) and amongst the 50 proteins with the most decreased (‘Top50 DOWN’) nascent levels in CO versus N were many important for splicing.

Search for interaction networks in the upregulated group (‘UP’ with log2 H/M > 0.6) further revealed enrichment in proteins related to essential metabolic processes important for the adaptation to hypoxic environment, like HIF1α signaling and heme biosynthesis (Figures 6F and 6G). The data indicated activation of pathways that are known to promote cancer cells survival such as autophagy, the anaplastic lymphoma kinase (ALK) (Figure 6G) as well as TGFβ signaling, as shown in (Figure 6B). Proteins downregulated (‘DOWN’ with log2 H/M <−0.6) in the BMM-mimicking setup, are involved in the regulation of translation, rRNA maturation, ribosome biogenesis, splicing and RNA binding, including the ARE-site binding proteins that regulate mRNA stability and decay (Figures 6E and 6F). These data indicate that the profound rearrangement of the translation machinery is related to a specific regulation of protein synthesis in CO versus N setups.

Altogether, our results show that existence of leukemia cells in different microenvironments (blood versus bone marrow) modifies the cellular proteome to enable adaptation of CML cells to the extracellular context. This could be a reason for therapy resistance of the cells.

### Knockdown of TIAR or FMRP affects adaptation of the nascent proteome to the microenvironment

**Figure 7:**
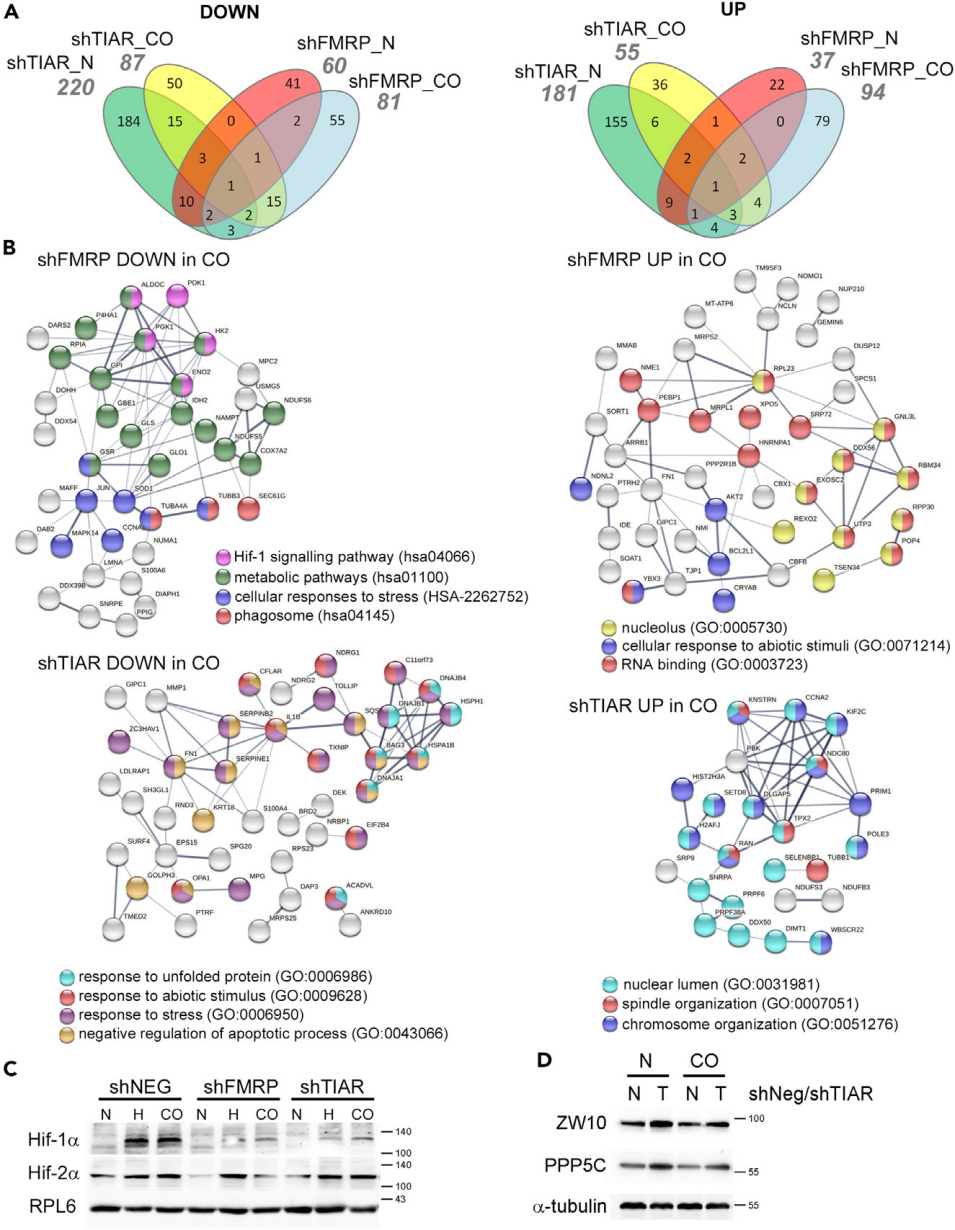
Modification of the nascent proteome by TIAR or FMRP silencing K562 cells transduced with shNEG, shTIAR or shFMRP (shRBP) were cultured for 72 h in N or CO before incorporation for 4 h of Met analog (L-HPG) and ‘heavy’ or ’medium’ Lys or Arg for QuaNCAT-MS. Results from 3 independent experiments. (A) Comparison of proteins upregulated (fold change log2>0.6, UP) or downregulated (log2<−0.6, DOWN) in shTIAR or shFMRP versus shNEG, in N or CO conditions. Total number of proteins in the group is in gray italic, and overlapping between the compared groups - in the Venns’ graphs. (B) Proteins exclusively regulated by shTIAR or shFMRP in CO condition analyzed for functional enrichment by string.db; proteins marked according to the legend below the graph; terms over-represented with Benj.Hoch. FDR≤0.05. (C and D) Protein level in total cellular extract analyzed by Western blotting.

Next, we verified whether TIAR and FMRP play a role in the observed proteome changes in response to microenvironment conditions. Using the QuaNCAT approach (Figure 6A) we identified from 2,493 to 2,708 proteins in each experimental setup and we quantified differences in the levels of nascent protein groups comparing cells with TIAR or FMRP knock-down, respectively, to shNEG transduced cells cultured in N or CO conditions (Figure 7A; list of proteins in Table S2). In general, shTIAR affected a much larger number of proteins than shFMRP and exerted a broader effect in N conditions, whereas shFMRP in the BMM mimicking setup. There are only a few proteins whose synthesis was changed in the given conditions by both shRBPs.

We then analyzed functional relevance (Figure S8) and networks formed by proteins regulated exclusively by each shRBP (Figure 7B). In the BMM-mimicking setup silencing of FMRP downregulated synthesis of proteins relevant for the Warburg effect, so the metabolic adaptation to microenvironment observed in CO setup (Figures 5D–5F) such as glycolysis, HIF-1 signaling and metabolism of reactive oxygen species. Moreover, shFMRP upregulated mainly RNA-binding- and nucleolar proteins involved in the rRNA processing and ribosome biogenesis. Of interest, proteins annotated to the same GO:BP category (but not the same proteins) were upregulated in N by shTIAR (see Figure S8A). Considering the impact exerted by the microenvironment (Figures 6D–6G), this implies that in respect to an adaptation of translation machinery, FMRP activity is relevant in the BMM setup, whereas TIAR in N.

In the bone marrow conditions (in relation to N), knock-down of TIAR downregulated proteins involved in the unfolded protein response and negative regulation of apoptosis but upregulated proteins of nuclear lumen and essential in cell cycle (spindle formation and chromosome organization) (Figure 7B).

Proteomic data indicated that silencing of either of the RBPs downregulated the synthesis of proteins involved in response to hypoxic conditions, confirmed by decreased level of HIF1α in cells (Figure 7C). The effect of shFMRP has never been shown, however, modulation of HIF1α translation at the ARE-site by TIAR was described elsewhere.^81^ This underlines the importance of TIAR and FMRP in the cellular adaptation to the BMM.

In addition, proteins affected by shTIAR irrespective of N or CO conditions were functionally related to chromosome segregation in cell cycle control (Figure S8B), which was further confirmed on the total protein level (Figure 7D). The Zeste White 10 (ZW10) is important for anaphase checkpoint^82^ and membrane trafficking ER-Golgi,^83^ whereas protein phosphatase 5 (PPP5C) regulates signaling cascades important in cancer cells such as TGFβ.^84^ The data indicate that TIAR activity could support therapy resistance and survival of leukemia cells in the bone marrow niche by exerting impact on the proteins that control proliferation and the stromal-leukemia cells crosstalk.

Notably, the effect of TIAR and FMRP exerted on the nascent proteome was highly modulated by the microenvironment and restricted to the particular subset of proteins. We referred to changes in the proteome between peripheral blood versus bone marrow mimicking conditions (CO to N) to the effect of silencing of particular RBP under BMM (comparing cells transduced with shTIAR or shFMRP to shNEG in CO) (Figure S9A). There were 1,316 proteins identified in CO/N and in at least two experiments in either shTIAR or shFMRP. This comparison showed that there are groups of proteins which synthesis is regulated by both TIAR and FMRP, so silencing of either of the RBP results in loss of the effect driven by BMM conditions compared to PBM. We searched for the proteins which mode of change CO/N has reverted on silencing of the particular RBP (Figure S9B), following the assumption that such dependence would occur if the RBP activity was essential for synthesis of the protein. We compared only the group which log2 value in CO/N comparison was >0.5 and <-0.5. Using this approach we noted proteins regulated exclusively by TIAR or FMRP and their activity could be detrimental in the processes identified through GO enrichment analysis (Figures 6D–6F and S8). We then searched in the human IRES Atlas database^85^ if there are putative IRES in the mRNAs corresponding to the proteins. This showed that synthesis rate of proteins with decreased level in CO relative to N but upregulated on shTIAR could be regulated by IRES (marked with a star in Figure S9B), and such tendency is not observed in case of shFMRP.

### Downregulation of TIAR but not FMRP modifies sensitivity of leukemia cells to homoharringtonine

**Figure 8:**
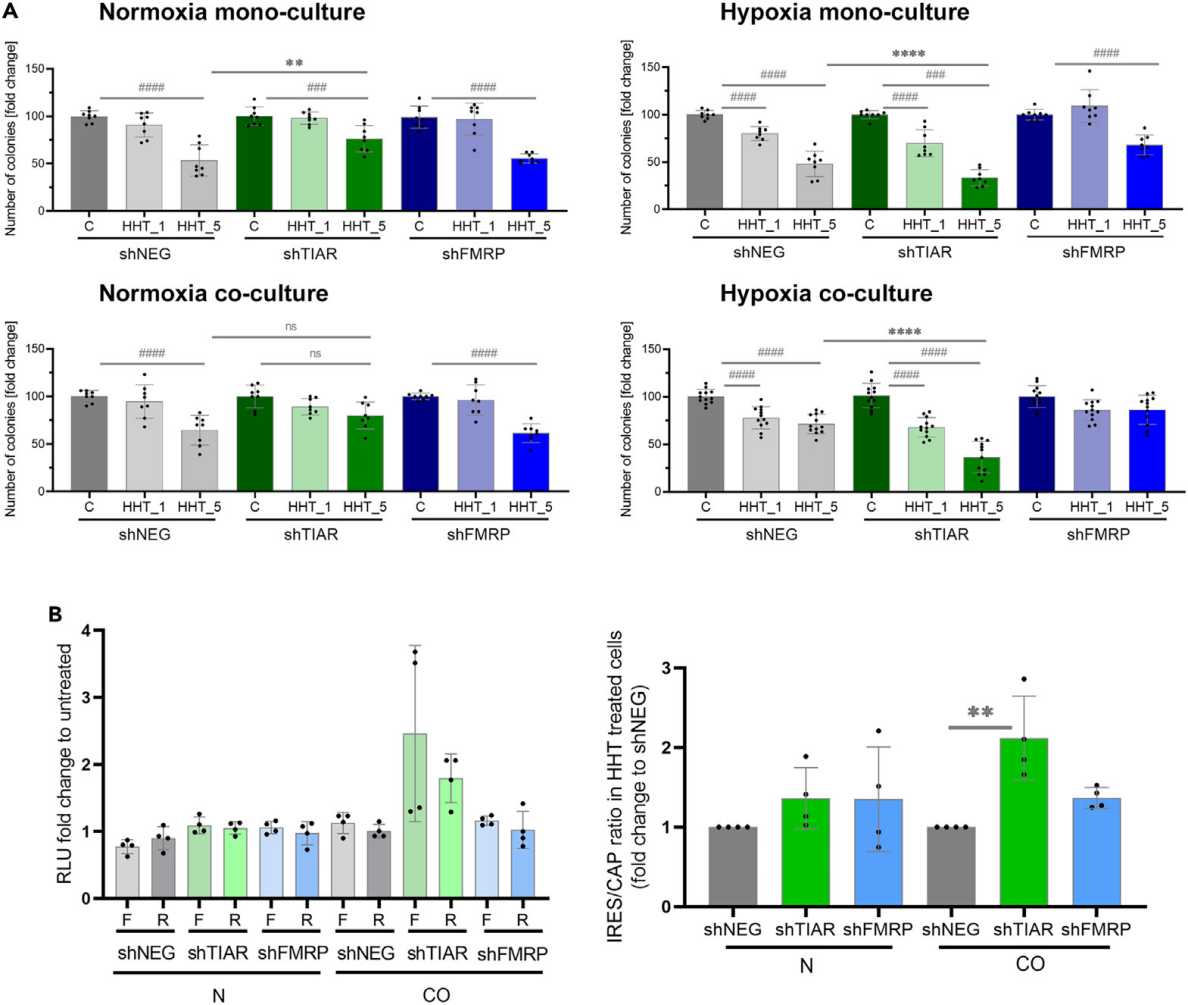
Impact of TIAR and FMRP silencing on the sensitivity to translation inhibitors The human K562 cells were cultured in normoxia or hypoxia, in mono-culture or co-culture with HS-5 cells. (A) Survival rate of leukemia cells transduced with shNEG, shTIAR or shFMRP analyzed based on colony forming after 72 h homoharringtonine treatment (HHT_1 – 1 nM and HHT_5 – 5 nM) added in 2 doses, at 0 and 24 h. (B) IRES- (F) and cap- (R) dependent translation (left panel) and ratio IRES/cap (right panel) tested by dual-luciferase assay upon treatment with 1 nM HHT for 24 h. (A-B) Fold change mean value ± ME is presented, n = 4 (replicates presented in A). Student’s *t*test was used to compare untreated with HHT treated cells (#) and one-way ANOVA to compare shTIAR with shNEG and shFMRP cells in HHT_5 cells; ∗p<0.05, ∗∗p<0.005, ∗∗∗p<0.001, ∗∗∗∗p<0.0001.

Our results demonstrated that the knock-down of TIAR or FMRP had influence on the translation and adaptation of the proteome to the hypoxic BMM conditions. Therefore, based on data presented by us and others, indicating that microenvironment-induced changes and remodeling of metabolome and proteome play critical role in the therapy resistance of CML cells,^8^^,^^86^ we hypothesized that targeting the translation process could eradicate leukemia cells from the bone marrow niche. To address this, we tested homoharringtonine (HHT, omacetaxine) that blocks translation elongation and is approved for treatment of CML patients.^87^ Because there is no report yet if the BMM (hypoxia and presence of stromal cells) exerts influence on the response of CML cells to HHT, we compared the inhibitor efficacy in normoxic and hypoxic conditions in mono- and co-culture (Figure 8A). To this end, we employed the colony-forming assay, which is an essential test for drug screening, and a model cell line.^5^^,^^8^^,^^50^ Treatment with HHT displayed no effect on the size of colonies formed (data not shown). Here we demonstrate that the hypoxic BMM could support resistance of leukemic cells to HHT and interaction with stromal cells played critical role in this respect. The effect of 5 nM HHT on control cells, transduced with shNEG, was much better pronounced in mono-culture than co-culture conditions (reduction by 44 ± 6% p<0.0001 and 52 ± 5% p<0.0001 versus 36 ± 6% p = 0.0002 and 29 ± 4% p<0.0001 in normoxia versus hypoxia, respectively). Thus the difference that could be attributed to the impact of interaction with stromal cells under 5 nM HHT is around 8% in normoxia and 23% in hypoxia. This comparison demonstrates that interaction of leukemia cells with stromal cells provides protective effect, additionally enhanced under hypoxia. Knock-down of FMRP affected sensitivity of cells to 5 nM HHT treatment only in a hypoxia mono-culture setup and caused reduction of the effect by 20 ± 6% p = 0.0049. Under hypoxia mono-culture effect of 5 nM HHT in shTIAR cells was enhanced (by 15 ± 6% p = 0.0208 comparing to shNEG-transduced cells, ANOVA one-way p<0.0001). In mono-culture under normoxia TIAR-depleted cells displayed lower sensitivity to 5 nM HHT (by 20 ± 7%, p = 0.0155 to the control cells, ANOVA one-way p = 0.0021). On the other hand, the data confirmed the critical role of TIAR in resistance of leukemia cells to HHT treatment in the hypoxic BMM-mimicking setup. In a co-culture setup silencing of TIAR significantly reduced the number of colonies formed on HHT in hypoxia (by 12 ± 4%, p = 0.0024 and 34 ± 8%, p<0,0001 to shNEG-transduced cells on 1 nM and 5 nM HHT, respectively; ANOVA one-way p<0.0001 in 5 nM HHT), whereas the difference was not significant in normoxia (by 5 ± 7%, p = 0.4482 and 15 ± 7%, p = 0.0555 to the control cells on 1 nM and 5 nM HHT, respectively). These data indicate that hypoxia could modulate the impact of TIAR on the sensitivity to treatment. In particular they point that protective impact of stromal cells on the effect of HHT treatment is provided under hypoxia and not normoxia and lack of TIAR diminishes this effect.

The changes in translation induced by TIAR silencing could constitute the basis of increased sensitivity to HHT in the BMM mimicking setup. Targeting of ribosome by HHT might stimulate IRES-dependent translation initiation.^75^^,^^88^ Because we observed that shTIAR increased the ratio of IRES/cap-dependent translation on hypoxia (Figure 5D), we determined how this would be affected by HHT (Figure 8B). In cells treated with HHT under BMM conditions silencing of TIAR significantly increased IRES/CAP ratio by 1.12 ± 0.26 p = 0.0054 in comparison to the ratio in shNEG-transduced cells (ANOVA one-way p = 0.0021 in BMM). Our results showed that increased sensitivity of shTIAR cells in CO versus N setup correlated with enhanced ratio of IRES/cap-dependent translation on HHT treatment in CO versus N. Considering that mRNAs encoding most of proteins upregulated in CO on shTIAR contain putative IRES (Figure S9B) this implicates that perturbance of selective protein synthesis might play a critical role in the support of leukemia resistance on the hypoxic BMM.

## Discussion

Interactions between cancer cells and their microenvironment are crucial for the development of leukemia. A variety of cytokines, growth factors, adhesion molecules and extracellular matrix proteins are secreted by both cancer and non-cancer cells, mediating cell-cell communication within the local microenvironment and providing a suitable niche for leukemia cells growth and survival.^89^^,^^90^ The role of stromal cells in protection of cancer cells from apoptosis has been identified in case of CML^56^^,^^57^ as well as AML.^91^ For instance, inhibition of the chemokine SDF-1 receptor CXCR4 in CML cells disrupts their direct interaction with the bone marrow niche cells what sensitizes them to BCR-ABL1 inhibitor.^1^^,^^92^ Furthermore, presented here proteomic data showed activation of TGFβR-induced signaling, and we had previously demonstrated support of CML and AML survival by this pathway in the context of hypoxic BMM.^5^ Various routes by which stromal cells can support the cancer cells have been uncovered. Apart from released factors, the protective effect provided by stromal cells could be also mediated by a direct cell-cell interaction involving tunneling nano-tubes formation^8^ or activation of surface receptors like Notch.^6^

The direct link between these routes of leukemia-stromal cells cross-talk and reduced formation of polyribosomes that we noted in co-culture conditions is yet to be determined. It might be related to the potentiation of eIF2α phosphorylation. There are data indicating a possible connection between stroma-provided signal and ISR. For instance, Notch1 directly regulates expression of genes encoding proteins of ERAD machinery (endoplasmic reticulum (ER)-associated protein degradation complex) essential for proteostasis in ER^93^ and thus induction of ISR. Stimulation of CXCR4 as well as TGFβR leads to activation of STAT3 signaling^94^^,^^95^ that triggers protein kinase R-like ER kinase (PERK),^96^^,^^97^ that phosphorylates eIF2α.^59^

Activation of stress response in leukemia cells seems to relate to adaptation to hypoxic bone marrow microenvironment, what is visible not only at the level of signaling proteins, but also when looking at the proteomic data. Induction of the Warburg effect by hypoxia evokes the metabolic switch and makes cancer cells more dependent on glycolysis in term of energy production.^9^^,^^10^ Here, we present that in the bone marrow versus blood-mimicking conditions enzymes participating in the glycolysis, oxidation-reduction potential, transport of oxygen and other proteins relevant for the HIF-1 response are upregulated. Of interest, such broad metabolic rearrangement was also noted in CML cells exposed to long-term imatinib treatment in normoxia and attributed to acquired resistance.^86^ Taken together, that would indicate that proteomic rearrangement on the BMM constitutes the bases for therapy resistance observed in this setup.

Our main finding is that remodeling of the translation process could be an important regulatory step involved in the cells’ physiology and therapy resistance in the hypoxic bone marrow niche. Moreover, we demonstrate that in response to the microenvironment the RNA binding proteins could have significant impact on the nascent proteome. Here we show that TIAR and FMRP modify synthesis of disparate sets of proteins that constitute cellular proteome in the given conditions. Thus, different cellular processes are modulated by their activity. Silencing of FMRP indicates its critical role in the metabolic functioning of the cells. That is in agreement with described observations.^47^^,^^98^^,^^99^^,^^100^^,^^101^ Besides, it enhanced synthesis of proteins involved in the ribosome biogenesis and rRNA processing. Knockdown of TIAR revealed impact of the protein on the stress response, survival of cells and regulation of cell cycle. The reduction of translation rate observed in the bone marrow conditions is an important feature of quiescent stem cells. In this aspect, the impact of FMRP and TIAR on the nascent proteome might play an important role in CML cells. It was postulated that the short isoform of Tia proteins could be important in establishing of quiescent phenotype,^102^ wide analysis showed importance of transcripts with AU-rich elements in chemoresistance of dormant AML cells,^103^ and role of FMRP granules was demonstrated in muscle stem cells.^104^ Because CML chemoresistance is attributed to acquiring of quiescence in the BMM,^1^ TIAR and FMRP activity could essentially modify leukemia cells’ sensitivity to therapy.

The RBPs-driven effects could result from direct interaction with selected transcripts or impact on the translation machinery. The straight effect of the RBPs would arise from the activity of TIAR and FMRP, because when bound to mRNA the RBPs orchestrate its accessibility to translation. The role of direct selective impact of RBPs supports the fact that our proteomic findings are consistent with published data showing that stress granules and P-bodies are enriched in mRNAs for proteins of distinct functionality.^46^ This may be a consequence of discrete target sequence recognition by the RBPs. Apart from mRNA binding and triage for translation, also interaction with translation machinery and complexing with ribosomal proteins could shape the profile of protein synthesis. We detected that in normoxia conditions FMRP is predominantly present in polyribosomes fraction, whereas silencing of FMRP enhanced formation of polyribosomes and accelerated recruitment of ribosomal proteins to the polyribosomes. This observation completes with data showing that FMRP can inhibit translation by interacting with polyribosomes and stalling of ribosomes on selected mRNAs.^75^^,^^105^ In case of shTIAR we observed reduced formation of polyribosomes. The same effect was observed on silencing of G3BP1,^106^ a stress granule protein like TIAR. The authors postulated that lack of association of G3BP1 and USP10 with ubiquitinated RPS2 and RPS3 in collided ribosomes restricts recycling of ribosomal subunits, that otherwise would signal for lysosomal degradation on ribosome-associated protein quality control (RQC).^107^ This sheds light why in our experiments reduction of polyribosomes on shTIAR was concomitant with reduced distribution of some ribosomal proteins to the polyribosomal fraction or enhanced presence in the monosome fraction.

Our results demonstrate that ribosomal proteins represent a significant pool of TIAR and FMRP interactors and this networking is responsive to the change of microenvironment. The selectivity of TIAR and FMRP interaction with ribosomal proteins is interesting in the view that silencing of either FMRP or TIAR had no essential effect on the general rate of proteins synthesis in the cells, however disturbed the profile of translation process and had selective impact on the nascent proteins. We did not found obvious correlation between interaction with particular RBP (IP results) and effect on distribution of the selected ribosomal proteins within the gradient on silencing of the RBP. Future, more in depth studies of the full RPs profile would allow us to determine effect of TIAR and FMRP on ribosomes composition and its implications. Ribosomal heterogeneity has been demonstrated to play a role in cancer development and therapy resistance.^108^^,^^109^^,^^110^ Noteworthy, point mutations in RPs contribute to development of leukemia, such as R98S in RPL10 in T cell ALL.^111^ In this view our observations indicate that TIAR and FMRP proteins could interact with some specialized ribosomes which convey translation at the edge of stress granules or p-bodies, however full understanding of this issue requires separate studies.

Importantly, we report for the first time that inhibition of translation by HHT in CML cells could be reduced in the bone marrow niche, and TIAR protein might play a role in this process. This finding might have a translational importance. Mechanism of HHT action is based on the targeting of ribosome active site and inhibition of translation elongation.^112^ Thus, efficiency of the drug might be compromised in a setup, where performance of the process of protein synthesis is generally reduced. Besides, such impact of BMM would be in agreement with its protective role displayed in case of treatment with imatinib^1^^,^^2^^,^^6^^,^^7^^,^^8^^,^^56^^,^^57^^,^^92^ or PARP1 inhibitors.^5^ Herein, we showed that silencing of TIAR sensitizes CML cells to HHT in the BMM. Because polyribosome profiling and analysis of RPs distribution in the profile did not show significant differences, therefore it is the change in translation of specific proteins on TIAR silencing that makes the cells more vulnerable to the translation inhibitor used. Of note is the response of TIAR-depleted cells to HHT induced IRES-dependent translation in CO. This suggests that modified selectivity of translation may cause the increased sensitivity to translation inhibition. Strikingly, most of proteins that were reversely upregulated on shTIAR contain putative IRES, whereas such correlation in case of FMRP is not observed. These proteins play crucial role in processes, which functioning could be changed on TIAR activity, based on the computational analysis of GO enrichment. The DDX50 RNA helicase could play a role in the ribosomal RNA synthesis and processing.^113^^,^^114^ The mitochondrial carrier homolog 2 (MTCH2) plays critical role in triggering apoptosis^115^ and determination of hematopoietic stem cells fate.^116^ The mitochondrial import inner membrane translocase subunit TIM50 (TIMM50) is a counterpart of complex that incorporates proteins into the interior of mitochondria and plays critical role in their functioning.^117^

In conclusion, we have discovered that the BMM, which include hypoxia and stromal component, significantly impacts the regulatory signaling and molecular counterparts of the translation machinery. The differences in translation between the blood and bone marrow are partly driven by hypoxia itself and presence of stromal cells displays synergistic effect. However, this work indicates that RNA binding proteins could induce specific proteomic adaptation enforced by particular microenvironment. Therefore, our data improved an understanding of the BMM-mediated changes in leukemia cells and might contribute to development of more effective therapeutic strategies.

### Limitations of the study

General overview and focus of our work is on the endpoint effect of translation regulation – level of newly synthesized proteins and their function. We aimed at comparing how proteome is adapted to see what functionalities could be changed in favor of the therapy resistance in the bone marrow microenvironment. It would be very interesting to study the RNA corresponding to the identified proteins. In the manuscript we show correlation between the higher proportion of monosomes with the reduced level of particular protein synthesis rate. Comparison of these data with such results obtained with the use of approach such as RiboSeq analysis of separated monosome and polysome fraction would enable to draw specific conclusions. Herein we checked IRES/Cap-dependent translation. There are number of mechanisms identified, such as RNA structure, ribosome stalling or NMDA, that could interfere with the process of mRNA translation and affect the protein synthesis level. Analysis of RNA would demonstrate the mechanisms involved in regulation of mRNA susceptibility for translation initiation and further steps.

We were interested if activity of RNA binding proteins changes profile of the nascent proteome. We observed that the RBPs interact with ribosomal proteins, however, we didn’t find any obvious correlation indicating dependence of RPs activity on interaction with TIAR or FMRP and their distribution in the gradient. But our analysis of ribosomal proteins was limited only to several proteins. For most of the RPs tested the change of % distribution in the mono-/polyribosomal fraction was accompanied by modified formation of mono-/polyribosomes. This would support the notion that protein composition of ribosomes is in fact not changing. Systematic comparison with the use of quantitative mass spectrometry of ribosomal proteins composition in each fraction would give better insights.

In most of the experiments we compared the effect evoked under hypoxia by stromal cells on leukemia cells to mono-culture of the leukemia in hypoxia, rather than co-culture in normoxia. The reason for this is that hypoxia itself exerts significant effect on the leukemic cells biology and we tested if interaction with stromal cells plays additive effect in this microenvironment. We show here that biology of leukemic cells is strongly affected by long-term hypoxia (elevated HIF1α, reduced population doubling and translation efficiency rate) and we described previously modified presence of surface receptors such as TGFβR.^5^ Therefore, signaling of stromal cells in hypoxia might evoke different effect than in non-hypoxic cells. Moreover, to gain precise insight into the impact of stromal cells on translation regulation other experimental designs ought to be used simultaneously. For instance, to study the role of direct cell-cell contacts a co-culture separated by a mesh should be employed. This could be supplemented by a setup based on leukemia cells mono-culture in conditioned medium from stromal cells to assay impact of released factors.

## STAR★Methods

### Key resources table

**Table:** 

REAGENT or RESOURCE	SOURCE	IDENTIFIER
**Antibodies**		

Mouse monoclonal anti-α-tubulin	Calbiochem	Cat#CP06-100UG
Mouse monoclonal anti-β-actin	Sigma Aldrich	Cat #A5316
Mouse monoclonal anti-eIF2α	CellSignaling	Cat #2103
Rabbit polyclonal anti-phospho Ser51-eIF2α	CellSignaling	Cat #9721
Rabbit monoclonal anti-eIF4E	CellSignaling	Cat #2067
Rabbit polyclonal anti-phospho Ser209-eIF4E	CellSignaling	Cat #9741
Rabbit polyclonal anti-FMRP	Bethyl	Cat#A305-199A
Mouse monoclonal anti-FXR1P	Santa Cruz Biotechnology	Cat#sc-374148
Mouse monoclonal anti-FXR2P	Santa Cruz Biotechnology	Cat #sc-376963
Rabbit polyclonal anti-GAPDH	Bethyl	Cat#A300-639A
Mouse monoclonal anti-G3BP1	Proteintech	Cat#66486-1-Ig
Rabbit monoclonal anti-HIF1α	CellSignaling	Cat#14179
Rabbit monoclonal anti-HIF2α	Bethyl	Cat#A700-003-T
Mouse monoclonal anti-HuR	Santa Cruz Biotechnology	Cat #sc-5261
Rabbit polyclonal anti-PABP	Proteintech	Cat#10970-1-AP
Rabbit monoclonal anti-PARP1	CellSignaling	Cat #9532
Rabbit monoclonal anti-PERK	CellSignaling	Cat#5683
Rabbit polyclonal anti-PPP5C	Proteintech	Cat #11715-1-AP
Rabbit polyclonal anti-phospho Ser371-p70S6K	CellSignaling	Cat #9208
Rabbit polyclonal anti-RPL6	Proteintech	Cat#15387-1-AP
Rabbit polyclonal anti-RPL10	CellSignaling	Cat#72912
Mouse monoclonal anti-RPL17	Proteintech	Cat#67223-1-Ig
Rabbit polyclonal anti-RPL18	Proteintech	Cat#17029-1-AP
Rabbit polyclonal anti-RPL21	Proteintech	Cat#15226-1-AP
Rabbit polyclonal anti-RPL28	Proteintech	Cat#16649-1-AP
Rabbit polyclonal anti-RPL31	Proteintech	Cat#16497-1-AP
Rabbit polyclonal anti-RPL37A	Proteintech	Cat#14660-1-AP
Rabbit polyclonal anti-RPS2	Abcam	Cat#ab264336
Mouse monoclonal anti-RPS3	Proteintech	Cat#66046-1-Ig
Mouse monoclonal anti-RPS6	Proteintech	Cat#66886-1-Ig
Rabbit polyclonal anti-RPS15	Proteintech	Cat#14957-1-AP
Rabbit polyclonal anti-Tia-1	Proteintech	Cat#12133-2-AP
Rabbit polyclonal anti-TIAR	Bethyl	Cat#A303-613A
Rabbit monoclonal anti-mTOR	CellSignaling	Cat#2983
Rabbit monoclonal anti-phospho Ser2448–mTOR	CellSignaling	Cat#5536
Mouse monoclonal anti-Smad3	Proteintech	Cat#66516-1-Ig
Rabbit monoclonal anti-phospho Ser423/425-Smad3	CellSignaling	Cat#9520
Rabbit polyclonal anti-ZW10	Proteintech	Cat#24561-1-AP
Goat anti-mouse/HRP secondary Ab	Dako	Cat#P0447
Goat anti-rabbit/HRP secondary Ab	Dako	Cat#P0448
Goat anti-rabbit IgG/StarBright Blue 700 secondary Ab	BioRad	Cat#12004162
Goat anti-mouse IgG/StarBright Blue 520 secondary Ab	BioRad	Cat#12005867
Rabbit monoclonal anti-HuR/Alexa Fluor 647	Abcam	Cat#ab209609
Purified rabbit isotype IgG	Bethyl	Cat#P120-101

**Bacterial and virus strains**		

MISSION® TRC2 pLKO.5-puro Non-Mammalian shRNA Control Transduction Particles	Merck	Cat#SHC202V
MISSION® shRNA Lentiviral Transduction Particles for FMRP knockdown: TRCN0000059759 (clone 1); TRCN0000286973 (clone 2); TRCN0000286972 (clone 3); TRCN0000294378 (clone 4); TRCN0000298271 (clone 5)	Merck	Cat#SHCLNV-M_002024
MISSION® shRNA Lentiviral Transduction Particles for TIAR knockdown: TRCN0000276257 (clone 1); TRCN0000276212 (clone 2); TRCN0000276240 (clone 3); TRCN0000276211 (clone 4); TRCN0000017212 (clone 5).	Merck	Cat#SHCLNV-M_003252

**Chemicals, peptides, and recombinant proteins**		

Homoharringtonine (HHT)	Selleckchem	Cat#S9015
Cycloheximide (CHX)	SigmaAldrich	Cat# 01810
L-homopropargylglycine (L-HPG)	Jena Bioscience	Cat#86256
L-azidohomoalanine	ThermoScientific	Cat#C10102
azide-PEG3-biotin	Jena Bioscience	Cat#86256
TAMRAlabeled azide	Jena Bioscience	Cat#CLK-FA008
RPMI-1640 Medium without methionine, cystine and L-glutamine	SigmaAldrich	Cat#R7513
FBS	Gibco	Cat#A33820-01
Fixable Viability Dye eFluor-780	ThermoScientific	Cat #65-0865-1
DMEM w/o L-Arg, L-Leu, L-Lys, L-Met	AthenaES	Cat#0421
L-Leu	AthenaES	Cat#0418
13C6, 15N2 L-Lysine	ThermoScientific	Cat#88209
13C6, 15N4 L-Arginine	ThermoScientific	Cat#89990
4,4,5,5-D4 L-Lysine	ThermoScientific	Cat#88437
13C6 L-Arginine	ThermoScientific	Cat#88210
tris(2-carboxyethyl)phosphine	SigmaAldrich	Cat#C4706
tris(3-hydroxypropyltriazolylmethyl)amine	SigmaAldrich	Cat#762342
Trypsin	Promega	Cat#V511A

**Critical commercial assays**		

NE-PER® Nuclear and Cytoplasmic Extraction Reagents	ThermoScientific	Cat#78833
Click-IT cell reaction buffer	ThermoScientific	Cat#C10269
Amaxa Cell line Nucleofector kit V	Lonza	Cat#VCA-1003
Dual-Luciferase Reporter Assay System	Promega	Cat#E1910
Passive Lysis Buffer	Promega	Cat#E1941

**Deposited data**		

ProteomeXchange Consortium via the PRIDE partner repository	This paper	PXD032332

**Experimental models: Cell lines**		

Human: K562	ATCC	Cat#CCL-243
Human: Kasumi-1	ATCC	Cat#CRL-2724
Human: HL-60	ATCC	Cat#CCL-240
Human: HS-4	ATCC	Cat#CRL-11882
Human: LAMA-84	DSMZ	Cat#ACC 168
Human: BV-173	DSMZ	Cat#ACC 20
Human: NALM-6	DSMZ	Cat#ACC 128
Murine: OP-9	ATCC	Cat#CRL-2749
Murine: 32Dc13	provided by Dr. S.L. McKenna (Keeshan et al., 2001)^118^	Cat# CRL-11346
Murine: 32D-BCR-ABL1+	provided by Dr. S.L. McKenna (Keeshan et al., 2001)^118^	N/A
Murine: WEHI-3	provided by Dr. S.L. McKenna (Keeshan et al., 2001)^118^	Cat# TIB-68™

**Recombinant DNA**		

pcDNA3 RLUC POLIRES FLUC	Poulin et al., 1998^119^	Addgene plasmid, #45642; RRID: Addgene_45642

**Software and algorithms**		

ImageJ	Schneider et al., 2012^120^	https://ImageJ.nih.gov/ij/
Vsn package		http://bioconductor.org/packages/release/bioc/html/vsn.html
WebPlotDigitizer version 4.5		https://automeris.io/WebPlotDigitizer/; RRID: SCR_013996
MORPHEUS Versatile matrix visualization and analysis software		https://software.broadinstitute.org/morpheus/; RRID: SCR_017386
MaxQuant v. 2.0.3.0	Cox and Mann, 2008^121^	http://www.maxquant.org/; RRID: SCR_014485
Perseus version 1.6.10.0	Tyanova et al., 2016^122^	https://maxquant.net/perseus/; RRID: SCR_015753
g:GOSt (g:Profiler)		https://biit.cs.ut.ee/gprofiler/gost; RRID: SCR_006809
STRING database		https://string-db.org; RRID: SCR_005223
ClueGO		RRID: SCR_005748
CluePedia 2018 at CytoScape v. 3.7.2		RRID: SCR_003032

**Other**		

AttractSPE™ Disks Bio	Affinisep	Cat#SPE-Disks-Bio-C18-100.T1.47.20
Pierce High Capacity NeutrAvidin agarose beads	ThermoScientific	Cat#29202

### Resource availability

#### Lead contact

Further information and requests for resources and reagents should be directed to and will be fulfilled by the lead contact, Paulina Podszywalow-Bartnicka (p.podszywalow@nencki.edu.pl).

#### Materials availability

This study did not generate new unique reagents.

### Experimental model and subject details

Human chronic myeloid leukemia cell line K562 (#CCL-243) (F-female), human acute myeloid leukemia cell line Kasumi-1 (#CRL-2724) (M-male), human acute promyelocytic leukemia cell line HL-60 (#CCL-240) (F), bone marrow stroma fibroblast cell line human HS-5 (#CRL-11882) (M) and murine OP-9 (#CRL-2749) (from embryo) were obtained from AmericanType Culture Collection (ATCC; RRID: SCR_001672); human chronic myeloid leukemia cell line LAMA-84 (#ACC 168) (F), human acute lymphoblastic leukemia cell lines BV-173 (#ACC 20) (M) and NALM-6 (#ACC 128) (M) were obtained from DSMZ-German collection (RRID: SCR_001711). 32D mouse progenitor cells (32D, #CRL-11346) and BCR-ABL1-expressing 32D cells (32D-BCR-ABL+) and WEHI cells were kindly provided by Dr. S.L. McKenna.^118^ The 32D cells have been authenticated using the FTA Sample Collection Kit for Mouse Cell Authentication Service at ATCC. All cell lines were routinely tested for mycoplasma using PCR-based approach. Human cells were cultured in RPMI-1640 (Biowest, France, #L0490), whereas murine cells in Iscove’s Modified Dulbecco’s Medium (Dulbecco, #30–2005) with 10% WEHI conditioned medium; either was supplemented with 100 U/ml penicillin, 100 μg/mL streptomycin (Biowest, #L0022), 2 mM L-Glutamine (Biowest, #X0550) and 10% FBS (v/v; Biowest, #S1810) as previously described.^7^ For 72 h co-culture, cells were seeded at 1:1 leukemia-to-stroma ratio with HS-5 cells (for human cells) or OP-9 (for murine cells). Cell cultures were grown under normoxia condition (mono-cultures) in CO_2_ incubator under 37°C, 5% CO_2_ and 21% O_2_ or under hypoxic conditions (mono-cultures or co-cultures) in *InVivo* 400 hypoxia workstation (Baker Ruskinn, UK) under 37°C, 5% CO_2_ and 1.5% O_2_. Experiments were initiated after 72 h adaptation period to the hypoxic conditions.

### Method details

**Flow cytometry analysis of co-cultured cells** was performed as we have previously described.^7^ Briefly, to distinguish K562 from HS-5 cells, the HS-5 cells were seeded and labeled with 15 μM CMAC (Thermo Fisher Scientific) and K562 with 10 μM CFSE (ThermoFisher Scientific) or the Cell proliferation dye eFluor670 (eBioscience, Invitrogen/ThermoFisher Scientific), according to the manufacturer’s protocols. Apoptosis was verified using the **Annexin V-PE/7-AAD ApoptosisDetection** Kit (BD Biosciences Pharmingen) in accordance with the manufacturer’s instructions. The percentage of Annexin V-positive cells was calculated in the population of CFSE positive cells. The mean ± SEM of three independent experiments are shown. **Cell cycle analysis** was performed according to.^123^ Cells were washed in PBS and fixed overnight in ice-cold 70% ethanol at −20°C. Then, the cells were washed in PBS, incubated in an extraction buffer (8 mM citric acid, 0.2 M Na_2_HPO_4_) for 5minat the room temperature, followed by a staining buffer (3.8 mM sodium citrate, 50 μg/mL propidium iodide and 50 μg/mL RNase A) for 30minat RT. The percentage of cells in each phase of the cell cycle was calculated using the Modfit LT Dna Analysis Software. FACS analyses were performed using BD LSRFortessa. Data were acquired using BD FACSDiva 6.2 and analyzed using FlowJo v10.7.2.

**Population doubling time** was assessed according to the growth rate (GR), so how many times the population doubled within the time of 72 h (t) based on the number of cells seeded (Nt) and 72 h later (N0), using the following equation:

$$GR=\frac{\ln\left(\frac{Nt}{N0}\right)}{t}$$

Then population doubling time (PD) was calculated: PD=ln⁡(2)/GR. Results expressed in hours from at least 4 experiments ± ME are presented.

#### Cell lysis and Western Blotting

For total cell lysates, cells were washed with phosphate-buffered saline prior to lysis at 95°C in SDS lysis buffer (50 mM Tris-HCl pH 6.8, 10% glycerol and 2% SDS) for 5 min. The lysates were passed 5 times through 1 mL insulin syringe (BD, #320911) and then centrifuged to remove the debris. Nuclear and cytoplasmic proteins were isolated using NE-PER Nuclear and Cytoplasmic Extraction Reagents (ThermoScientific, #78833) according to the protocol provided. The same amount of proteins were mixed with the 5xSB buffer (250 mM Tris-HCl pH 6.8, 10% SDS, 50% glicerol, 0.15% bromophenol blue, 500 mM DTT), boiled at 95°C for 5 min and subjected to SDS-PAGE followed by Western Blotting analysis. Antibodies used to develop the Western blots were: anti-ATF4 (Proteintech, #10835-I-AP), anti-α-tubulin (Calbiochem, #CP06-100UG), anti-β-actin (Sigma Aldrich, #A5316), anti-eIF2α (CellSignaling, #2103), anti-phospho Ser51-eIF2α (Cell Signaling, #9721), anti-eIF4E (Cell Signaling, #2067), anti-phospho Ser209-eIF4E (Cell Signaling, #9741), anti-FMRP (Bethyl, #A305-199A), anti-FXR1P (Santa Cruz Biotechnology, #sc-374148), anti-FXR2P (Santa Cruz Biotechnology, #sc-376963), anti-GAPDH (Bethyl, #A300-639A), anti-G3BP1 (Proteintech, #66486-1-Ig), anti-HIF1α (Cell Signaling, #14179), anti-HIF2α (Bethyl, #A700-003-T), anti-HuR (Santa Cruz Biotechnology, #sc-5261), anti-PABP (Proteintech, #10970-1-AP), anti-PARP1 (CellSignaling, #9532), anti-PPP5C (Proteintech, #11715-1-AP), anti-phospho Ser371-p70S6K (CellSignaling, #9208), anti-RPL6 (Proteintech, #15387-1-AP), anti-RPL10 (Cell Signaling, #72912), anti-RPL17 (Proteintech, #67223-1-Ig), anti-RPL18 (Proteintech, #17029-1-AP), anti-RPL21 (Proteintech, #15226-1-AP), anti-RPL28 (Proteintech, #16649-1-AP), anti-RPL31 (Proteintech, #16497-1-AP), anti-RPL37A (Proteintech, #14660-1-AP), anti-RPS2 (Abcam, #ab264336), anti-RPS3 (Proteintech, #66046-1-Ig), anti-RPS6 (Proteintech, #66886-1-Ig), anti-RPS15 (Proteintech, #14957-1-AP), anti-Tia-1 (Proteintech, #12133-2-AP), anti-TIAR (Bethyl, #A303-613A), anti-mTOR (CellSignaling, #2983), anti-phospho Ser2448–mTOR (CellSingaling, #5536), anti-Smad3 (Proteitech, #66516-1-Ig), anti-phospho Ser423/425-Smad3 (CellSignaling, #9520), anti-ZW10 (Proteintech, #24561-1-AP), and secondary antibodies: conjugated with horseradish peroxidase (HRP) goat anti-mouse (Dako, #P0447) and goat anti-rabbit (Dako, #P0448) or StarBright Blue 700 conjugated goat anti-rabbit IgG (Bio-Rad, #12004162) and StarBright Blue 520 conjugated goat anti-mouse IgG (Bio-Rad, #12005867). Incubation with primary antibodies was carried in 5% bovine serum albumin (Sigma, #A3059) or non-fat milk in TBST buffer (20 mM Tris-HCl, 136 mM NaCl pH 7.6 with 0.1% Tween 20) overnight at 4°C with agitation. After 3 washes in TBST (each for 10minat room temperature) secondary antibodies were incubated for 1hat room temperature with agitation. Activity of HRP was assayed using ECL substrate (BioRad, #170–5061 and #170–5062 for weak signal). Western Blots were imaged and analyzed using ChemiDoc MP Imaging system (BioRad, RRID: SCR_019037). The intensity of the signal from Western Blot analysis was assessed using ImageJ software (RRID: SCR_003070)^120^ and function ‘Analyze – Gel’ and then normalized to the signal of loading control (β-actin, α-tubulin, PARP1 or GAPDH – depending on the type of experiment). The values for control samples constituted 100% and other variants were calculated accordingly.

#### Immunostainings

For immunostaining, cells were fixed with 3.7% paraformaldehyde in PBS for 10minat room temperature (RT) and permeabilized with cold 0.08% Triton X-100 for 5minat RT, followed by 30 min of blocking in 5% goat serum and 1 h staining with an anti-HuR primary antibody conjugated with Alexa Fluor 647 (Abcam, #ab209609) and diluted 100x in 0.5% goat serum and 0.05% Tween 20 in PBS. After three washing steps in 0.05% Tween 20 (Bioshop, #TWN510) in PBS, cells were stained with 0.1 μg/mL DAPI (#D9542, SIGMA) for 20 min. The coverslips were then washed five times in PBS and once in MilliQ water and mounted (Dako, #S3025). Analysis was performed at the Laboratory of Imaging Tissue Structure and Function (Nencki Institute) using Leica SP8 confocal microscope with a 60x immersion objective. Pixel size 50 × 50 μm and z-stacks of 0.50 μm thickness were collected. Line profile and 3D visualization in Arivis Vision4D ×64 3.5.1 (arivis AG) software (RRID: SCR_018000). Graphs prepared using GraphPad Prism version 9.3.1 (RRID: SCR_002798).

#### Immunoprecipitation

In order to immobilize antibodies used for immunoprecipitation, the mixture of 5 μg of anti-FMRP (Bethyl, #A305-199A) or anti-TIAR (Bethyl, #A303-613A) or rabbit isotype IgG (Bethyl, #P120-101) antibodies, 40 μL of A/G agarose beads (Santa Cruz Biotechnology, # 2003) and 750 μL of PBS buffer was incubated for 4 h on a rotor at 4°C and then washed three times in PBS. Cells were lysed in mild lysis buffer allowing the extraction of cytoplasmic proteins and containing 10 mM HEPES pH 7.2, 150 mM KCl, 5 mM MgCl_2_, 0.1% NP40, protease inhibitor cocktail (Roche, #11836153001), 1 mM NaF, 1 mM Na_3_VO_3_, 1 mM PMSF, 40 U/μL RiboProtect Hu (Blirt, #RT36) and DEPC-treated MilliQ water. Then 1000 μg of protein extracts were incubated overnight at 4°C with an indicated antibody-protein A/G agarose beads complexes, followed by washing three times with PBS. Protein complexes were detached from beads by adding 50 μL of 5xSB buffer, boiling and centrifugation at 16000xg for 10minat 37°C. Supernatants were transferred to a new tubes and subjected to Mass Spectrometry or Western Blot analysis.

**Mass Spectrometry Analysis of IP complexes** combined with tandem mass tagging (**TMT-MS**). Three biological replicates of cytoplasmic protein complexes immunoprecipitated with specific (α-TIAR, α-FMRP) or rabbit isotype IgG (procedure described in section ‘Immunoprecipitation’) were sent to the Proteomics Core Facility at EMBL Heidelberg, Germany, and analyzed according to the following protocol (presented on the Scheme in Figure 2A): each protein sample was subjected to isobaric labeling and then six samples from the given biological replicate were combined into one sample analyzed by LC-MS/MS in a single run. For the analysis of obtained proteomics data GNU R programming language was used. Only proteins that were quantified with two unique peptide matches were subjected to analysis. Additionally, proteomics data from isotype IgG were used as a background control. The vsn package from Wolfgang Huber was used to apply a variance stabilization normalization method on the log2 raw data. A protein was considered significant, if the adjusted p values (the false discovery rate adjustment for p values from Benjamini and Hochberg was used) was below 0.05 and a fold change of at least 50% was observed.

#### Monosome/polyribosome fractionation

Polyribosome Fractionation was performed as described in.^124^ Briefly, day before cell lysis, 10–50% sucrose gradients were prepared using GB buffer (10 mM HEPES KOH pH 7.2; 150 mM KCl; 5 mM MgCl_2_; 10 mM NaF; Protease Inhibitor Cocktail (Roche); 100 μg/mL CHX; 4U/ml RIBOProtect (Blirt) and DEPC water). Cells adapted to N, H or CO conditions were re-plated at the same density, keeping the corresponding conditions (HS-5 cells for CO seeded day before). The next day cells were treated with 100 μg/mL CHX and 12-15x10^6^ of cells was subjected to cell lysis in the GB buffer with 0.1% NP40 for 10 min on ice. After centrifugation at 18000xg for 5minat 4°C, the protein concentration in the supernatant was measured on NanoDrop, then the same amount of proteins was loaded on top of the gradient and spun using Optima XPN ultracentrifuge (RRID: SCR_018238) and SW41Ti rotor (Beckman Coulter) for 2hat 4°C, 38000 rpm (250000xg max). After centrifugation, the Density Gradient Fractionation System (Teledyne ISCO) with Foxy Jr. Fraction Collector was used to acquire sucrose fractions, stored for later usage at −80°C. During collection the absorbance of samples was monitored at λ = 254 nm and recorded to create profile of RNA distribution along the gradients, that was printed on the ISCO paper. The profiles were scanned and digitalized using WebPlotDigitizer version 4.5 (RRID: SCR_013996; by Ankit Rohatgi available at https://automeris.io/WebPlotDigitizer). Area under the peaks was analyzed using ImageJ software. For the assessment of ribosomal proteins (RPs) abundance in the sucrose fractions, 30 μL of each fraction was mixed with 6 μL of the 5xSB buffer, boiled and subjected to SDS-PAGE followed by Western Blotting analysis and densitometry of the signal. The sum of signal intensity on the blot for bands from each fraction was referred to as 100% and % of the band signal intensity for particular fraction was calculated.

#### Analysis of RNA abundance in sucrose fractions

For the analysis of RNA abundance, 200 μL of each sucrose fraction was transferred to a new 1.5 mL Eppendorf tube and 800 μL of TRIzol Reagent was added (ThermoScientific, #15596018). RNA extraction was performed using The Total RNA mini kit (A&A Biotechnology, #031–100) according to the manufacturer protocol. The same volume of eluted RNA was mixed with 2x loading buffer (ThermoScientific, #R0641) and subjected to electrophoresis in the 1% agarose gel with formaldehyde in TBE buffer, stained with ethidium bromide and visualized under UV. ChemiDoc Imaging System (BIO-RAD) to archive the RNA distribution.

#### Heat maps

All heat maps were prepared using MORPHEUS Versatile matrix visualization and analysis software (RRID: SCR_017386; https://software.broadinstitute.org/morpheus/). For mass spectrometry proteomics data log2 of fold change values were used to draw heat maps.

#### Lentiviral transduction

For stable gene knockdown, the MISSION shRNA lentiviral particles from Merck/Sigma Aldrich (St. Louis, MO, USA) were used. Briefly, 8 × 10^3^ of K562 cells were cultured in 96-well plates, incubated for 2hat 37°C, and then 5 different lentiviral particles were added at MOI of 5. After incubation of cells for 72hat 37°C, 1 μg/mL puromycin was added. To establish stable knockdown cell lines, clones 1–5 were subjected to selection for additional 14 days in medium containing 1 μg/mL puromycin and pooled. The level of gene silencing was verified with RT-qPCR and Western Blot analysis. MISSION shRNA lentiviral particles used: MISSION TRC2 pLKO.5-puro Non-Mammalian shRNA Control Transduction Particles (SHC202V); MISSION shRNA Lentiviral Transduction Particles for FMRP knockdown (SHCLNV-NM_002024): TRCN0000059759 (clone 1), TRCN0000286973 (clone 2), TRCN0000286972 (clone 3), TRCN0000294378 (clone 4), TRCN0000298271 (clone 5); for TIAR knockdown (SHCLNV-NM_003252): TRCN0000276257 (clone 1), TRCN0000276212 (clone 2), TRCN0000276240 (clone 3), TRCN0000276211 (clone 4), TRCN0000017212 (clone 5).

#### Measurement of general translation efficiency using Click-IT AHA

The efficiency of general protein synthesis was measured using Click-IT AHA technique (ThermoScientific) accordingly to the protocol provided by the producer. Briefly, cells were washed in warm PBS twice and cultured for 1hat 37°C in cell culture medium w/o methionine and cysteine: RPMI-1640 (SigmaAldrich, #R7513) supplemented with 10% of filtered FBS (Gibco, #A33820-01), and 2 mM L-Glutamine (BioWest, #X0550). Then L-azidohomoalanine (Thermo Fisher, #C10102) was added to a 25 μM final concentration with or without 100 nM HHT. After 4 h incubation at 37°C, cells were washed twice in cold PBS and stained with eBioscience Fixable Viability Dye eFluor-780 (ThermoScientific, #65-0865-14) for 30 min on ice and protected from light. After two washing steps with cold PBS, cells were subjected to 15 min fixation with 4% formaldehyde and then 15 min permeabilization in 0.25% Triton X-100 in PBS. Click-IT REACTION MIX consisting of 1x Click-IT cell reaction buffer (ThermoScientific, #C10269), CuSO_4_, Click-IT cell buffer additive and alkyne Alexa Fluor 488 (ThermoScientific, #A10267) was prepared just before the use. Cells were washed with 3% BSA in cold PBS and 250 μL of Click-IT REACTION MIX was added followed by 30 min incubation on ice in the dark. After indicated time, two washing steps with 3% BSA in cold PBS were performed, then cells were re-suspended in 250 μL of cold PBS and intensity of fluorescence was measured on LSRFortessa (BD). Only viable cells were analyzed.

#### IRES/cap-dependent translation

In order to study impact of regulatory elements on translation effectiveness a bicistronic vector was used. The pcDNA3 RLUC POLIRES FLUC was a gift from Nahum Sonenberg (Addgene plasmid, # 45642; http://n2t.net/addgene:45642; RRID: Addgene_45642).^119^ Translation of *Renilla* luciferase was cap-dependent, whilst synthesis of firefly luciferase was directed by the poliovirus IRES and is therefore cap-independent. 1.2 × 10^6^ of 72 h adapted cells (N, H, CO or NCO condition) were subjected to nucleofection with 1 μg of plasmid using the Amaxa Cell line Nucleofector kit V (Lonza, #VCA-1003) and Amaxa Nucleofector 2b (Lonza), according to the manufacturer protocol. The experimental procedure was performed under normoxia or hypoxia, respectively. Following the nucleofection, the cells were seeded back in the corresponding experimental setting N, H, CO or NCO (HS-5 cells were seeded a day before) in a 6-well plate. After 24 h the cells were lysed in the Passive Lysis Buffer (Promega, #E1941) for 10minat RT and activities of the enzymes were measured sequentially using the Dual-Luciferase Reporter Assay System (Promega, #E1910) and the Glomax 20/20 luminometer (Promega). Then the ratio of obtained relative luminescence units (RLU) for firefly to *Renilla* luciferase was calculated and used to assess the efficiency of cap-dependent and IRES-dependent translation fold change.

**Treatment with translation targeting inhibitor, homoharringtonine** (**HHT**; CAS No. 26833-87-4; from Selleckchem, #S9015) was used as a positive control in experiments aiming to measure the efficiency of general translation with the use of Click-IT AHA and flow cytometry (after 4 h of treatment) as well as the IRES/Cap-dependent translation (after 24 h). To this end, the 50 mM stock solution in dimethyl sulfoxide (DMSO; SIGMA #4540) was diluted first in DMSO to 2 mM and then in RPMI medium to 100 μM or 1 μM to reduce concentration of DMSO solvent (dilution in medium was prepared each time, on the day of addition to the cells). Dependently on the experiment and final concentration required, 1 μL of the medium-diluted 100 μM or 1 μM HHT was added to 1 mL of cell culture for a final concentration of 100 nM or 1 nM HHT, respectively. Apart from that, the impact of homoharringtonine was tested using a colony forming assay. In this case, the 2 mM HHT in DMSO was diluted in the RPMI medium to 600 nM HHT, of which 0.5 μL or 2.5 μL was added to 300 μL of cell culture (grown in 48-well plate #353078 Corning) for a final concentration of HHT 1 nM or 5 nM, respectively. Further details relating HHT treatment regime are provided with the description of the colony forming assay.

#### QuaNCAT sample preparation

To study differences in the level of nascent peptides synthesis the cells were incubated with 1 mM L-homopropargylglycine (L-HPG) (Jena Bioscience, #86256) for 4hat 37°C in DMEM w/o L-Arg, L-Leu, L-Lys, L-Met (AthenaES, #0421), supplemented with 10% of filtered FBS (Gibco), 2 mM L-Glu (Biowest), 0.8 mM L-Leu (AthenaES, #0418), and supplemented with isotypically labeled L-Lys (0.67 mM) and L-Arg (0.40 mM) to label the newly synthesized peptides as ‘heavy’: 13C_6_, 15N_2_ L-Lysine (#88209) and 13C_6_, 15N_4_ L-Arginine (#89990); or as ‘medium’: 4,4,5,5-D_4_ L-Lysine (#88437) and 13C_6_ L-Arginine (#88210); all from ThermoScientific. After two washing steps with cold PBS, cells were lysed in 2% SDS in PBS for 5 min in RT and stored at −80°C prior further analysis. Lysates from equal counts of the ‘heavy’ and the ‘medium’ cells were combined. The combined lysates were sonicated and diluted to final concentration (f.co.) of 0.5% SDS in PBS. The samples were subjected to the click reaction with 0.1 mM azide-PEG_3_-biotin (Jena Bioscience, #86256) in the click reaction mixture containing: 1 mM CuSO_4_, 2 mM tris(2-carboxyethyl)phosphine (TCEP; SigmaAldrich, #C4706) and 0.4 mM tris(3-hydroxypropyltriazolylmethyl)amine (THPTA; SigmaAldrich, #762342) for 1hat 25°C with 1100 rpm shaking. Then the reaction was quenched by adding EDTA to 5 mM f. co., followed by precipitation of proteins with methanol (1:1, v/v) and chloroform (0.25:1, v/v). Precipitated proteins were collected at 3500xg for 5minat RT, the precipitates sonicated in 1 mL of methanol, spun at 18000xg for 5minat RT and after 3 rounds of this methanol addition/sonication/centrifugation/methanol removal protocol the proteins were air dried and solubilized in 2% SDS in PBS, sonicated and diluted with PBS to 0.5% SDS. Then 30 μL of the Pierce High Capacity NeutrAvidin agarose beads (ThermoScientific, #29202) were added to each sample (washed in 0.5% SDS/PBS) and incubated for 2hat 25°C with 1100 rpm shaking. After that, the beads were subject to a series of washing and centrifugation at 5000xg for 3minat RT: 3x with 0.5% SDS/PBS, 3x with PBS, 2x with 100 mM ammonium bicarbonate/H_2_O (AMBIC). The beads were suspended in AMBIC supplemented with 5 mM TCEP and 10 mM chloroacetamide. Trypsin (Promega, #V511A) was added (1 μg/sample with 800 μg of input protein) and the beads were incubated at 37°C overnight with 1100 rpm shaking. The beads were pelleted at 5000xg for 3 min and the supernatant containing peptides was collected, acidified with trifluoroacetic acid to f.co. 0.5% and stored at −80°C. The supernatants were then desalted with the use of AttractSPE Disks Bio – C18 (Affinisep, # SPE-Disks-Bio-C18-100.T1.47.20) using a published stage-tip protocol,^125^ and concentrated using a Savant SpeedVac concentrator. Prior to LC-MS measurement, the samples were re-suspended in 0.1% TFA, 2% acetonitrile in water.

#### LC-MS/MS analysis of QuaNCAT samples

Chromatographic separation was performed on an Easy-Spray Acclaim PepMap column 50 cm long × 75 μm inner diameter (ThermoScientific) at 45°C by applying a 150 min acetonitrile gradients in 0.1% aqueous formic acid at a flow rate of 300 nL/min. An UltiMate 3000 nano-LC system was coupled to a Q Exactive HF-X mass spectrometer via an easy-spray source (all ThermoScientific). The Q Exactive HF-X was operated in data-dependent mode with survey scans acquired at a resolution of 120,000 at m/z 200. Up to 12 of the most abundant isotope patterns with charges 2–5 from the survey scan were selected with an isolation window of 1.3 *m*/*z* and fragmented by higher-energy collision dissociation (HCD) with normalized collision energies of 27, while the dynamic exclusion was set to 30 s. The maximum ion injection times for the survey scan and the MS/MS scans (acquired with a resolution of 30,000 at m/z 200) were 45 and 150 ms, respectively. The ion target value for MS was set to 3e6 and for MS/MS to 2e5, and the minimum AGC target was set to 3e3.

#### Data processing for QuaNCAT samples

The data were processed with MaxQuant v. 2.0.3.0 (RRID: SCR_014485),^121^ and the peptides were identified from the MS/MS spectra searched against the reference human proteome UP000005640 (Uniprot.org) using the build-in Andromeda search engine. Raw files corresponding to 3 replicate samples obtained from K562 cells cultured for 72 h in N and CO conditions were processed together. Raw files corresponding to 3 replicate samples obtained from the cells subjected to four pairs of conditions (1. shNEG and shTIAR-transduced cultured in N; 2. shNEG and shTIAR-transduced cultured in CO; 3. shNEG and shFMRP-transduced cultured in N; 4. shNEG and shFMRP-transduced cultured in CO) were processed together in a separate search. During the primary search the multiplicity was set to 3 (Light labels: Lys0 & Arg0; Medium labels: Lys4 & Arg6; Heavy labels: Lys8 & Arg10) and the re-quantify function in the software disabled. During the secondary search the multiplicity was set to 2 (Medium labels: Lys4 & Arg6; Heavy labels: Lys8 & Arg10) and the re-quantify function enabled (this search was perform to allow inclusion in the analysis of proteins, for which either ‘Medium’ or ‘Heavy’ counterparts were not detected. Cysteine carbamidomethylation was set as a fixed modification and methionine oxidation, glutamine/asparagine deamidation, and protein N-terminal acetylation were set as variable modifications. For in silico digests of the reference proteome, cleavages of arginine or lysine followed by any amino acid were allowed (trypsin/P), and up to two missed cleavages were allowed. The FDR was set to 0.01 for peptides, proteins and sites. Match between runs was enabled. Other parameters were used as pre-set in the software. Unique and razor peptides were used for quantification enabling protein grouping (razor peptides are the peptides uniquely assigned to protein groups and not to individual proteins). Data were further analyzed using Perseus version 1.6.10.0 (RRID: SCR_015753)^122^ and Microsoft Office Excel 2016.

#### Analysis data processing and bioinformatics

Normalized H/M ratios for protein groups obtained from the primary/secondary MaxQuant search were loaded side-by-side into Perseus v. 1.6.10.0. Standard filtering steps were applied to clean up the datasets: reverse (matched to decoy database), only identified by site, and potential contaminant (from a list of commonly occurring contaminants included in MaxQuant) protein groups were removed. The H/M ratios were log2 transformed and protein groups with log2 H/M values in at least 2 out of 3 replicates were kept. The protein groups obtained from the primary search were added the pre-filtered protein groups obtained from the secondary search (all proteins within the added protein groups of the secondary search had to be absent in protein groups of the primary search). The data in the combined datasets were normalized by subtraction of median H/M values for each sample. Protein groups for which all the detected Log2 H/M values were >0, and at least two Log2 H/M values were >0.6 were classified as upregulated in H. Protein groups for which all the detected Log2 H/M values were <0, and at least two Log2 H/M values were < −0.6 were classified as upregulated in M. Annotation enrichment analysis of proteins upregulated in H or upregulated in M was performed using g:GOSt (g:Profiler RRID: SCR_006809, https://biit.cs.ut.ee/gprofiler/gost) with Benjamin-Hochberg FDR set to 0.05. Analysis of protein networks enrichment was done by search in the STRING database (RRID: SCR_005223, https://string-db.org) and ClueGO (RRID: SCR_005748) with CluePedia 2018 at CytoScape v. 3.7.2 (RRID: SCR_003032) with Benjamini-Hochberg FDR set to 0.05. The Human IRES Atlas database (http://cobishss0.im.nuk.edu.tw/Human_IRES_Atlas/) (Yang et al., 2021) was used to check for putative IRES structures.

#### Protein degradation/stability rate

The cells were in N, H or CO setup for 48 h and then 3x10ˆ6 of cells were incubated with 0.1 mM L-HPG similarly like for QuaNCAT sample preparation for 4hat 37°C in 3 mL of DMEM w/o L-Met but with 0.8 mM L-Leu, 0.67 mM L-Lys and 0.40 mM L-Arg (AthenaES, #0421), supplemented with 10% of filtered FBS and 2 mM L-Glu. Before and after labeling the cells were washed once in PBS (added in 10:1 v/v to cell culture) and centrifuged in RT, 160xg for 3 min (shorter than for regular cultivation). Then 1.8x10ˆ6 cells were suspended in 12 mL of the complete growth medium and portions of cells were collected for cell lysis after: 16 h, 24 h and 40 h, whilst 1.2x10ˆ6 of cells were lysed immediately (as 0 h time point). Cells were lysed in 2% SDS in PBS for 5 min in RT and stored at −80°C prior further analysis. Prior the experiment all the media and PBS used were kept in the incubator or hypoxia workstation in vented flasks for at least 1 h to equilibrate.

#### Visualization of nascent proteins by in-gel fluorescence

A fraction of QuaNCAT cell lysates (50 μg of protein) was utilized to visualize nascent peptides by in-gel fluorescence. To this end, the click reaction with 0.1 mM TAMRA-labeled azide was performed (Jena Bioscience, #CLK-FA008) for 1hat 25°C in the click reaction mixture (see above). Following addition of EDTA and precipitation of proteins with methanol/chloroform the precipitates were sonicated in methanol, pelleted by centrifugation at 3500xg for 5minat RT and air-dried. The protein pellets were dissolved in sample loading buffer and boiled for 5 min. Equal protein amounts of samples were loaded onto 10% polyacrylamide gels and separated by SDS-PAGE. In-gel fluorescence signal of TAMRA was read prior to 1 h fixation (50% methanol/10% glacial acetic acid), 20 min staining with 0.1% Coomassie Brilliant Blue R-250 in the fixation buffer and de-staining (40% methanol/10% glacial acetic acid). ChemiDoc Imaging System (BIO-RAD) was used to image the gels. To monitor degradation rate the fluorescence intensity signal from each, entire length row was subjected to densitometry analysis (measured as one band) using ImageJ software. Signal from TAMRA was normalized to Coomassie. Then the 0 h time point was used as 100% and consecutive time points (16, 24 and 40 h) demonstrate level of the normalized signal as % of the signal in 0 h.

#### Colony forming assay

Colony forming assay was performed as previously described.^5^ Briefly, HS-5 cells were seeded 48 h before addition of K562, then first dose of homoharringtonine at 1 nM or 5 nM final concentration was added the next day and second dose the following day. After another 24 h the cells were seeded in methylcellulose-based medium (StemCell, #H4230) supplemented with 10% FBS. The colonies formed by the cells that survived the treatment were counted after 5 days.

#### Schemes

Schematic demonstration of experimental workflow as well as Venn’s graphs presented in figures were created with biorender.com.

### Quantification and statistical analysis

All procedures were executed for three independent biological experiments (n), unless stated otherwise. In case of each experiment of the colony formation assay the cells were grown in replicates or triplicates (black dot on the graphs in Figure 8A). Data were presented as the mean ± ME. Statistical analysis was performed using GraphPad Prism version 9.3.1 (RRID: SCR_002798). The unpaired, two-tailored Student’s *t* test was used to compare two samples with the same variation. One-way ANOVA was used to compare three groups of samples. The results with p ≤ 0.05 were considered statistically significant and designated by: ∗p ≤ 0.05; ∗∗p ≤ 0.01; ∗∗∗p ≤ 0.001; ∗∗∗∗p ≤ 0.0001.

## Data Availability

The proteomic data from this publication have been deposited to the ProteomeXchange Consortium via the PRIDE partner repository - identifier PXD032332, accessible with username: reviewer_pxd032332@ebi.ac.uk, password: 4gHQMNoy. This paper does not report original code. Any additional information required to reanalyze the data reported in this paper is available from the lead contact upon request.

## Supplemental information

Document S1. Figures S1–S9

Table S1. Proteins identified by QuaNCAT-MS in K562 cells in CO versus N conditions, related to Figure 6 K562 cells were cultured for 72 h in normoxia mono-culture (N) or hypoxia co-culture (CO) followed by incorporation into the synthesized peptides L-homopropargylglycine (L-HPG) and ‘heavy’/’medium’ Lys or Arg for 4 h. Click chemical reaction used to covalently bind biotin from azide to HPG, then nascent peptides enriched by binding to beads. Identification and quantification of the peptides using SILAC-MS based on the ratio of ‘heavy’ to ‘medium’ peptides intensity detected. Results from 3 independent experiments. Comparison of proteins upregulated (fold change log2>0.6, UP) or downregulated (log2<−0.6, DOWN) in CO versus N conditions.

Table S2. Proteins identified by QuaNCAT-MS in K562 cells transduced with shTIAR or shFMRP, related to Figure 7 K562 cells transduced with shNEG, shTIAR or shFMRP (shRBP) were cultured for 72 h in normoxia mono-culture (N) or hypoxia co-culture (CO) followed by incorporation into the synthesized peptides L-homopropargylglycine (L-HPG) and ‘heavy’/’medium’ Lys or Arg for 4 h. Click chemical reaction used to covalently bind biotin from azide to HPG, then nascent peptides enriched by binding to beads. Identification and quantification of the peptides using SILAC-MS based on the ratio of ‘heavy’ to ‘medium’ peptides intensity detected. Results from 3 independent experiments. Comparison of proteins upregulated (fold change log2>0.6, UP) or downregulated (log2<−0.6, DOWN) in shTIAR or shFMRP versus shNEG, in N or CO conditions. Presented is list of proteins (given by Gene ID) identified in the 4 compared samples (y), restricted to those that were UP/DOWN in at least two experiments, whereas the direction of change in the third experiment was remained or not detected.
