# Supplementary material for: TIAR and FMRP shape pro-survival nascent proteome of leukemia cells in the bone marrow microenvironment
Source: iScience. 2023 Mar 31;26(4):106543. doi: 10.1016/j.isci.2023.106543 (PMC10140000; doi:10.1016/j.isci.2023.106543)
Supplement: Document S1. Figures S1–S9 [file mmc1.pdf]

iScience, Volume *26*

Supplemental information

TIAR and FMRP shape pro-survival nascent proteome of leukemia cells in the bone marrow microenvironment

Magdalena Wolczyk, Remigiusz Serwa, Agata Kominek, Agata Klejman, Jacek Milek, Marta Chwałek, Laura Turos-Korgul, Agata Charzyńska, Michal Dabrowski, Magdalena Dziembowska, Tomasz Skorski, Katarzyna Piwocka, and Paulina Podszywalow-Bartnicka

S4SUPPLEMENTAL INFORMATION

SUPPLEMENTAL FIGURES

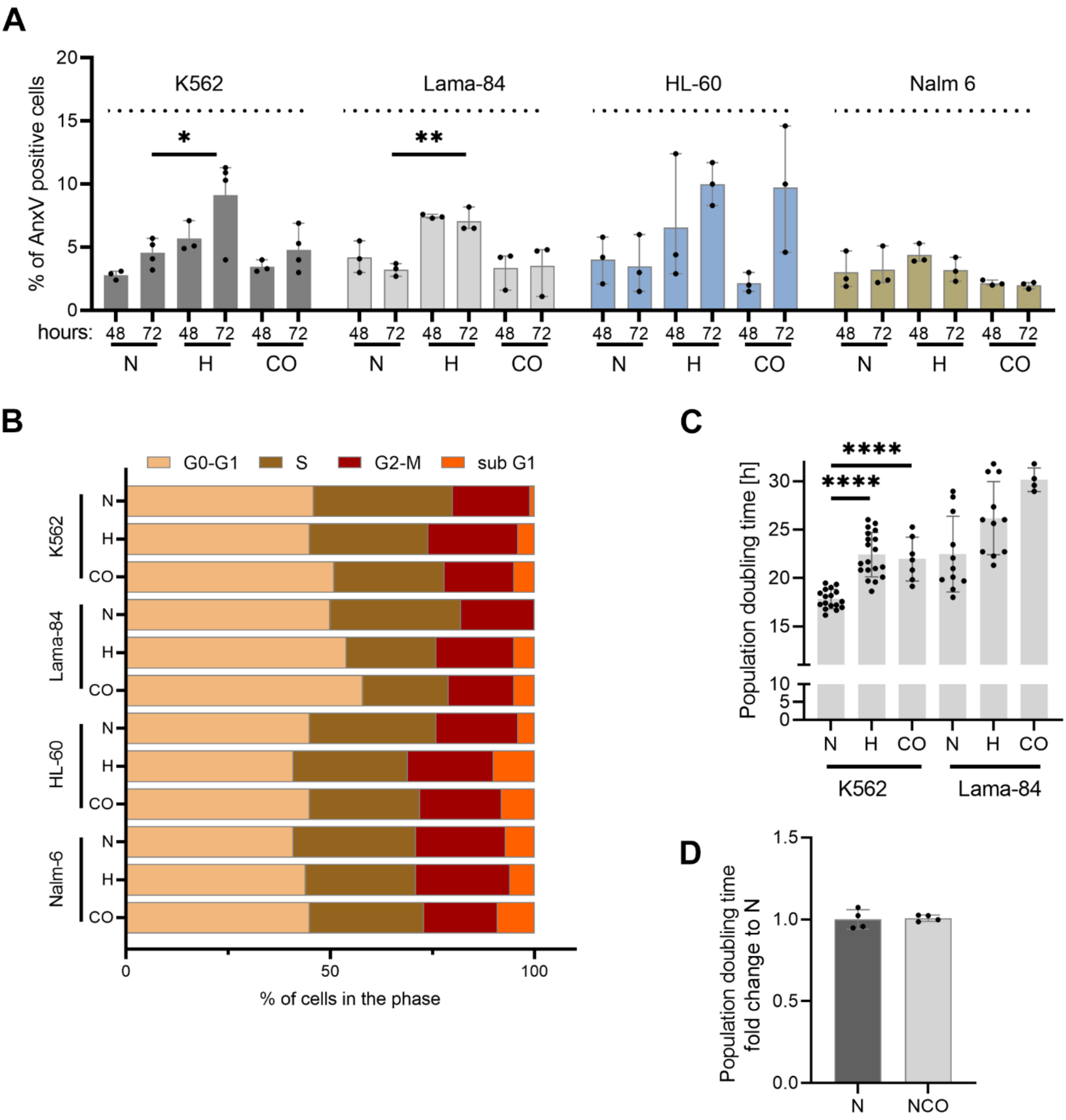

Figure S1. Effect of in vitro experimental setup that reflects blood and bone marrow like conditions on cell viability and cell cycle control of used cell lines, related to Figure 1. Various human leukemia cell lines (K562, Lama-84, HL-60 and Nalm6) were tested to determine the effect of mono-culture in normoxia (N), hypoxia (H) or co-culture with bone marrow stroma HS-5 (human) cell line under hypoxia (CO) or normoxia (NCO). After 72h the cells were analyzed by flow cytometry to check **(A)** level of apoptosis with AnxA5 and **(B)** cell cycle. **(C-D)** Population doubling time calculated based on growth rate, in **(D)** presented as fold change of NCO to N in K562 cells. Mean values obtained from at least three independent experiments (indicated by number of dots) ±ME are shown. Student's t-test was used to compare cells in N with H or CO; *p<0.05, **p<0.005, ****p<0.0005.

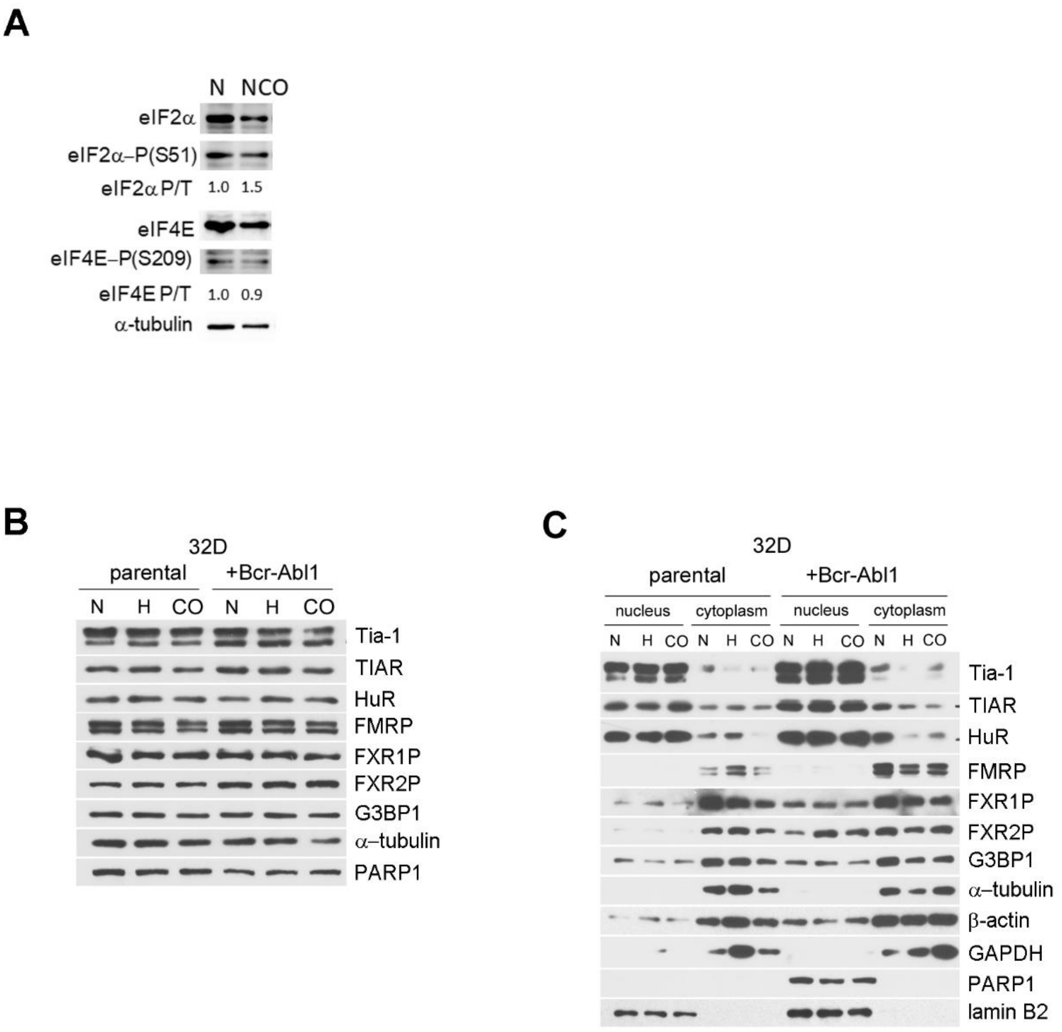

Figure S2. Impact of experimental setup in normoxia on translation eucaryotic initiation factor (eIF) phosphorylation, related to Figure 1. The human K562 cells were cultured in mono-culture (N) or co-culture with bone marrow stroma HS-5 cells (NCO) for 72h. Level of eIF was verified by Western blotting. Densitometry of signal ratio of phospho to total (P/T) is presented, normalized to α-tubulin and expressed as fold change of N. **(B-C) Impact of blood and bone marrow like conditions on the level and nucleo-cytoplasmic shuttling of selected RNA binding proteins in mouse CML cell line model.** Mouse (32D parental and with expression of *bcr-abl1* oncogene) were tested to determine the effect of mono-culture in normoxia (N), hypoxia (H) or co-culture with bone marrow stroma OP-9 (mouse) cell line under hypoxia (CO). Western blotting analysis of selected RNA binding proteins in **(B)** total cellular extracts and **(C)** cellular fractions of nucleus and cytoplasm.

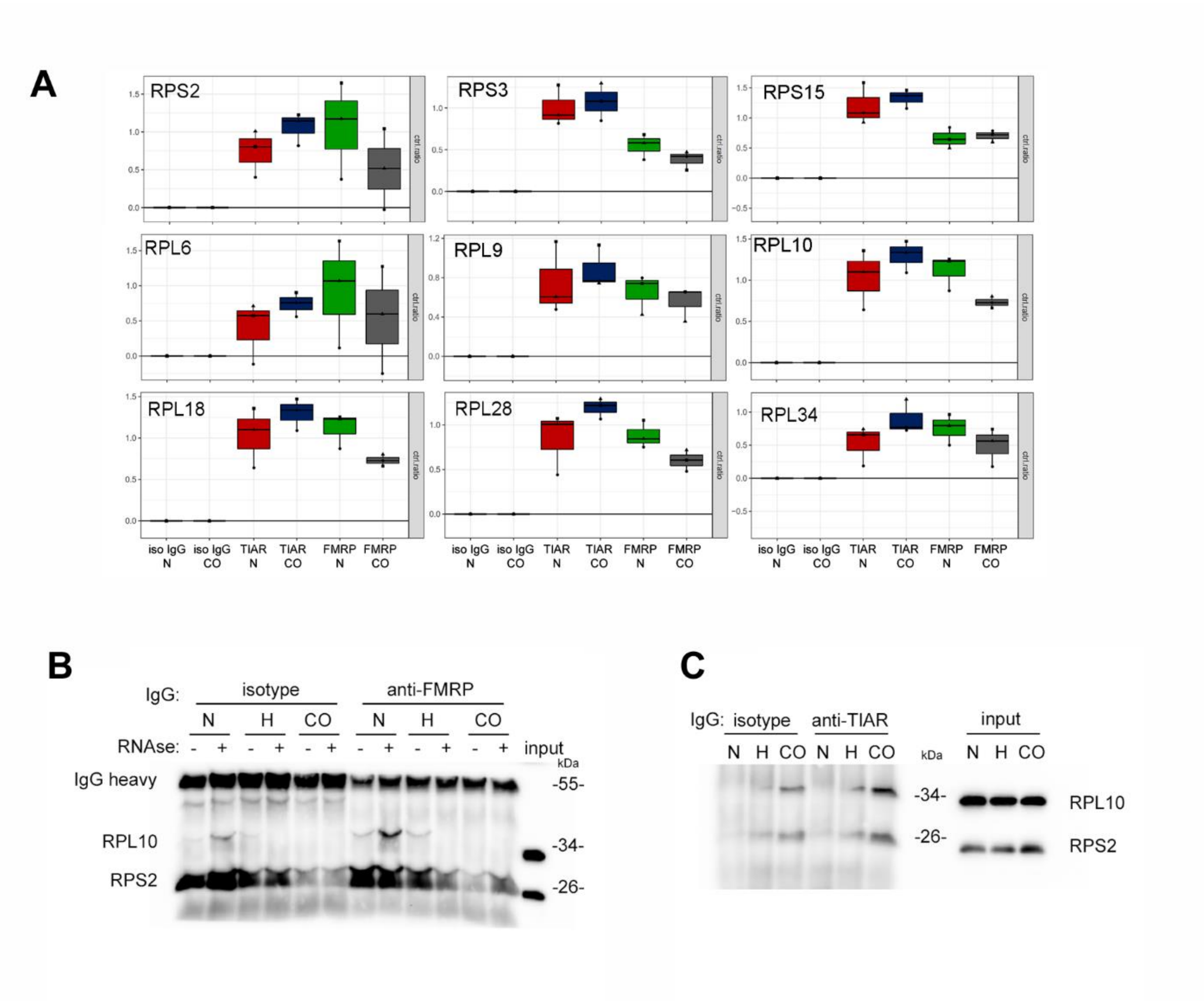

Figure S3. Microenvironment-dependent profile of proteins present in cytoplasmic complexes of TIAR and FMRP proteins, related to Figure 2. (A) Mass spectrometry analysis combined with tandem mass tagging (TMT-MS) was used to identify and quantify differences in the proteome of complexes immunoprecipitated from cytoplasmic fraction of K562 cells cultured either in normoxic mono-culture (N) or hypoxic co-culture (CO) for 72h. Profile of proteins isolated by immunoprecipitation (IP) with antibody of the same isotype (iso IgG) as the specific antibody used for IP of TIAR or FMRP was used as a background control. In total 6 isolations from each condition and IP were combined for isobaric labeling and analyzed by mass spectrometry in a single run. Graphs presenting normalized logFC values of selected ribosomal proteins identified with TMT-MS analysis. Data from 3 independent experiments are shown. **(B-C)** Western blotting analysis of RPL10 and RPS2 in IP complexes of iso IgG and FMRP **(B)** or TIAR **(C)**.

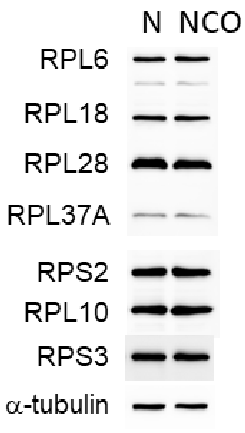

Figure S4. Level of selected ribosomal proteins analyzed by Western blotting in cell lysates loaded on the sucrose gradient (input) for polysome profiling, related to Figure 3. The human K562 cells were cultured in normoxia in mono-culture (N) or co-culture with bone marrow stroma HS-5 cells (NCO) for 72h.

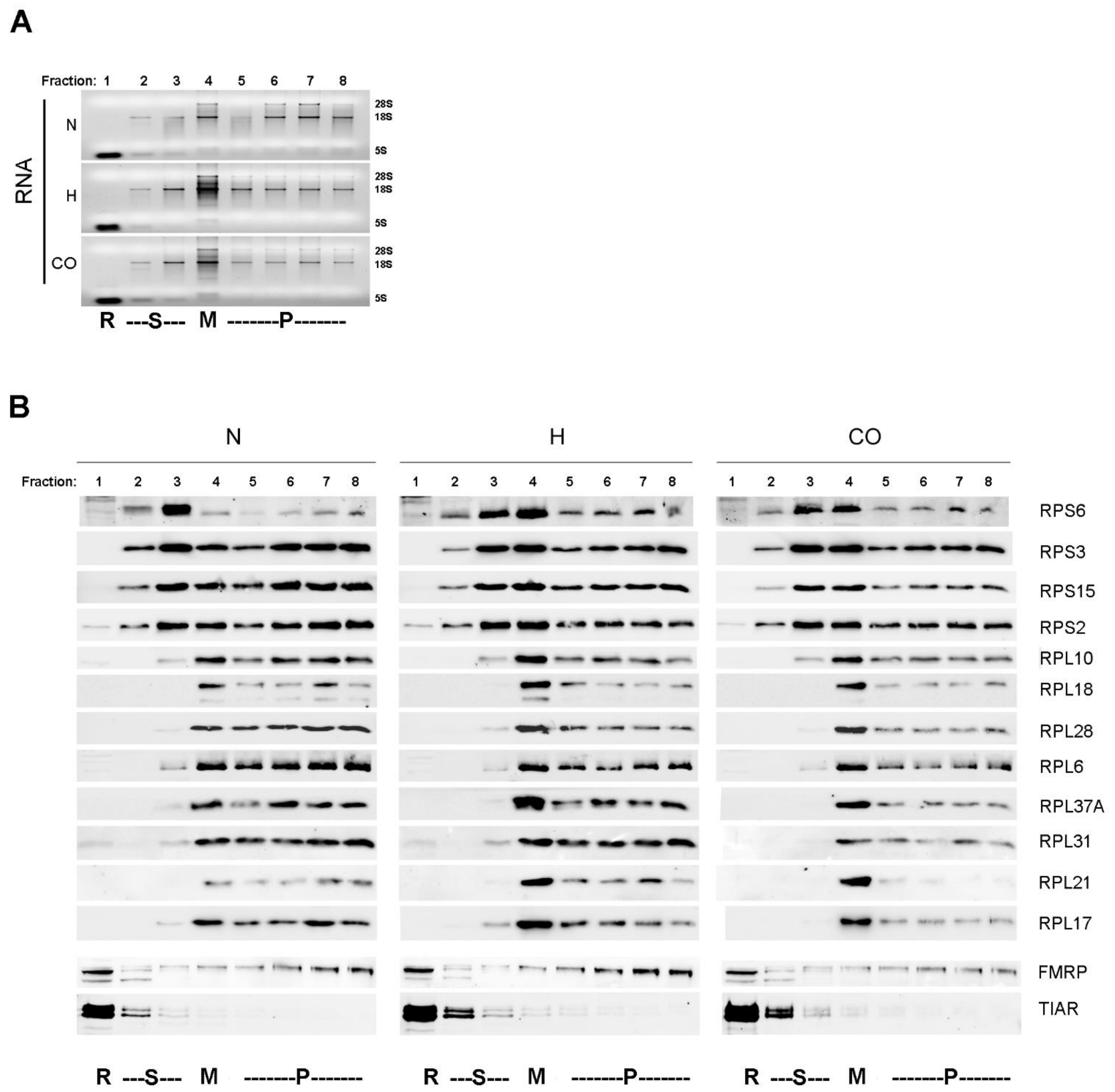

Figure S5. Microenvironment effect on the ribosomal proteins level in polysomal fractions, related to Figure 4. The human K562 cells were cultured in mono-culture in normoxia (N), hypoxia (H) or co-culture with bone marrow stroma HS-5 cells under hypoxia (CO) for 72h. Polyribosomes were separated by sucrose gradient ultracentrifugation and sucrose fractions (1-8) were collected from top to bottom of the gradient. The same volume of each fraction was used for analysis of RNA and protein distribution in the gradient. Representative images from 3 independent experiments are presented. **(A)** RNA in the agarose gel visualized under UV. Size of 28S, 18S and 5S rRNA indicated on the right. **(B)** Western blotting analysis of ribosomal proteins, FMRP and TIAR. Fractions denoted: R – RNA binding proteins complexes; S – small ribosomal subunit; M – large ribosomal subunit and monosome; P – polyribosomes.

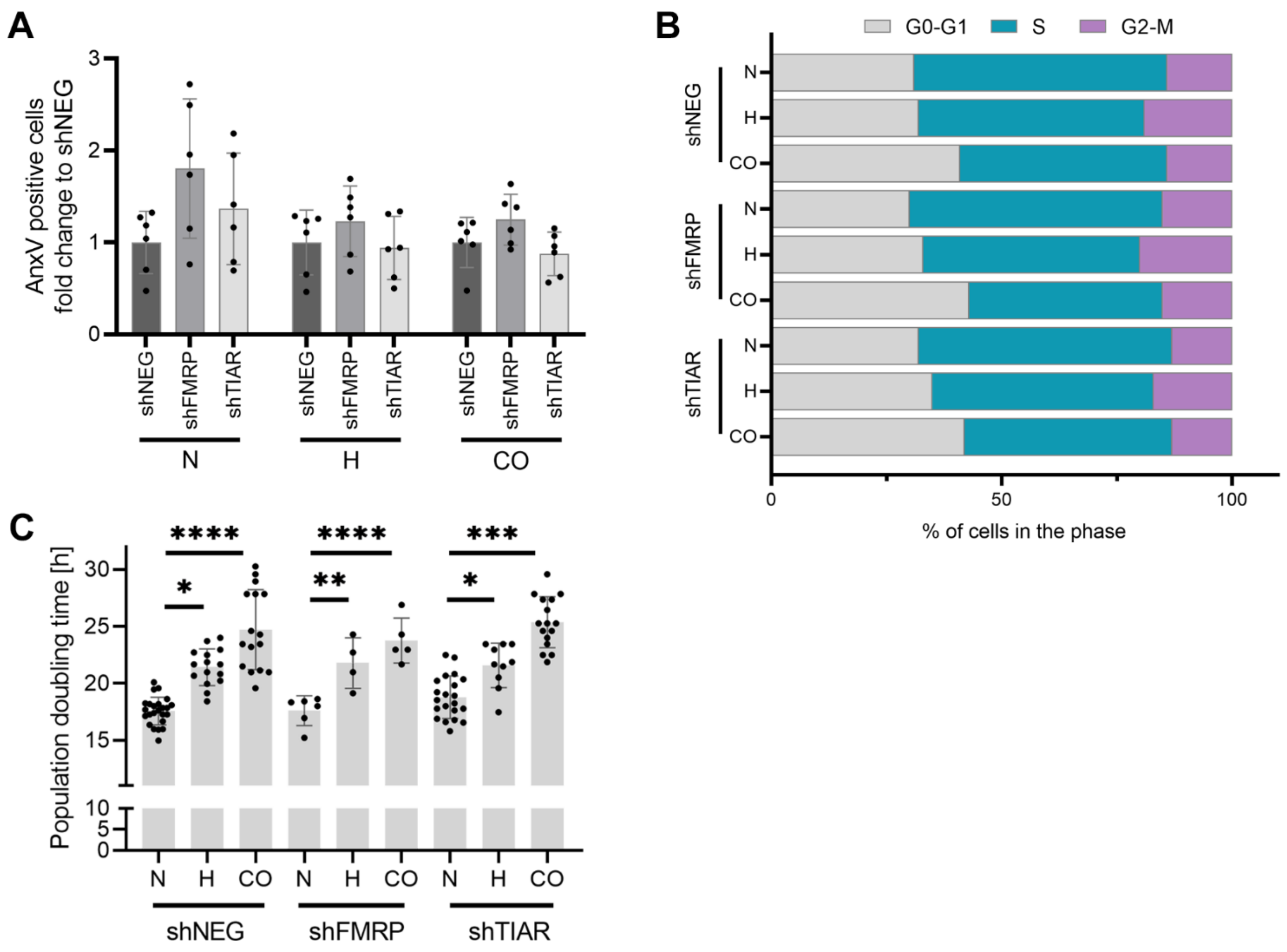

Figure S6. The effect of microenvironment conditions combined with FMRP or TIAR knock-down on the cells viability, cell cycle control and population doubling, related to Figure 5. The human K562 cells transduced with lentiviruses bearing negative shRNA (shNEG), shRNA targeting FMRP (shFMRP) or TIAR (shTIAR) were cultured in mono-culture in normoxia (N), hypoxia (H) or co-culture with bone marrow stroma HS-5 cells under hypoxia (CO) for 72h. After 72h the cells were analyzed by flow cytometry to check **(A)** level of apoptosis with Anx5 and **(B)** cell cycle. **(C)** Population doubling time calculated based on growth rate. Mean values obtained from three independent experiments ±ME are shown. Student's t-test to compare cells in N with H or CO; *p<0.05, **p<0.005, ****p<0.0005.

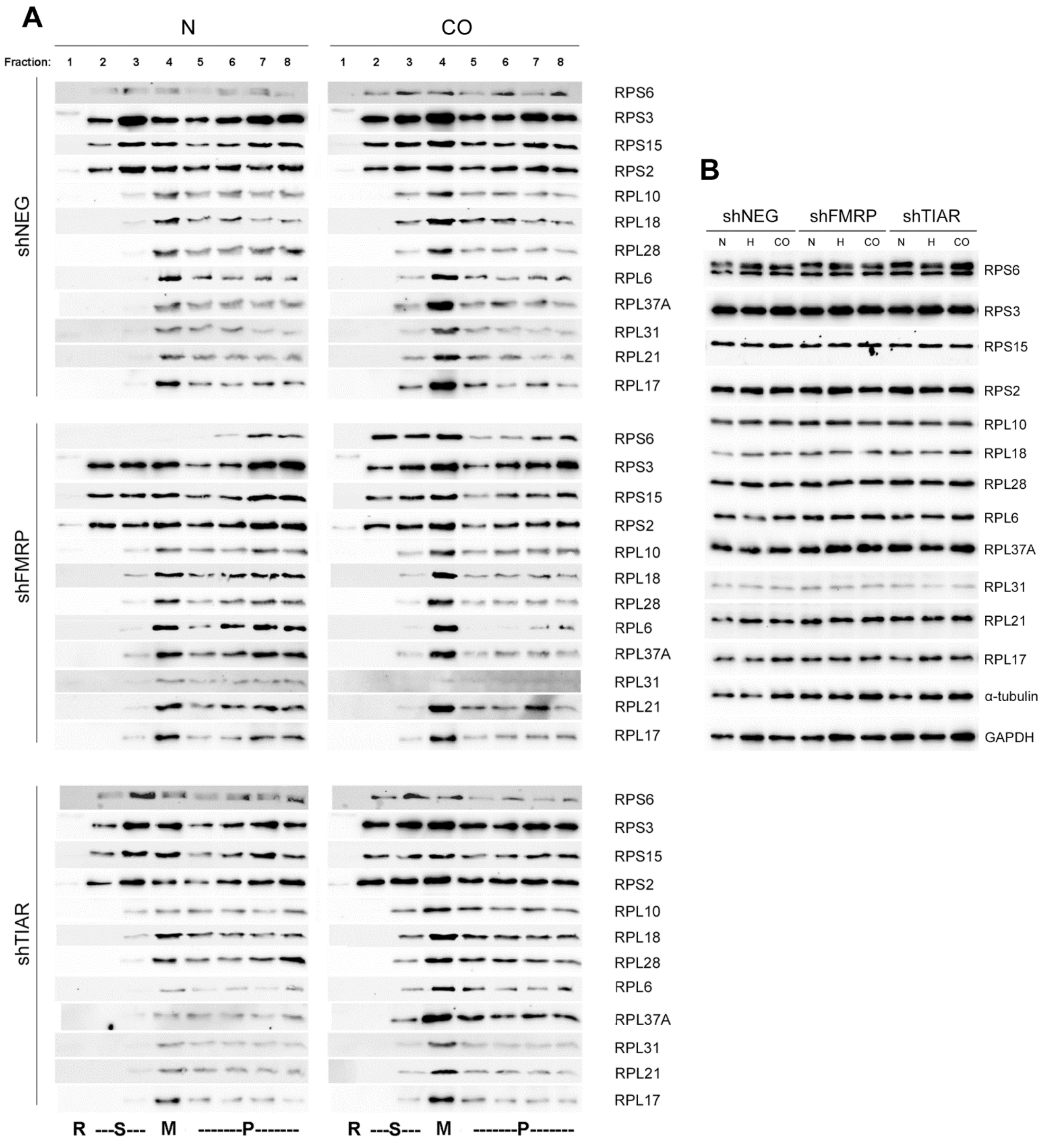

Figure S7. The effect of microenvironment conditions combined with FMRP or TIAR knock-down on the level of selected ribosomal proteins in polysomal fractions and cellular lysates,related to Figure 5. The human K562 cells transduced with lentiviruses bearing negative shRNA (shNEG), shRNA targeting FMRP (shFMRP) or TIAR (shTIAR) were cultured in mono-culture in normoxia (N), hypoxia (H) or co-culture with bone marrow stroma HS-5 cells under hypoxia (CO) for 72h. Level of selected ribosomal proteins analyzed by Western blotting in **(A)** polysomal fractions (1-8); the same volume of each fraction loaded; **(B)** cellular extracts loaded on the gradient.

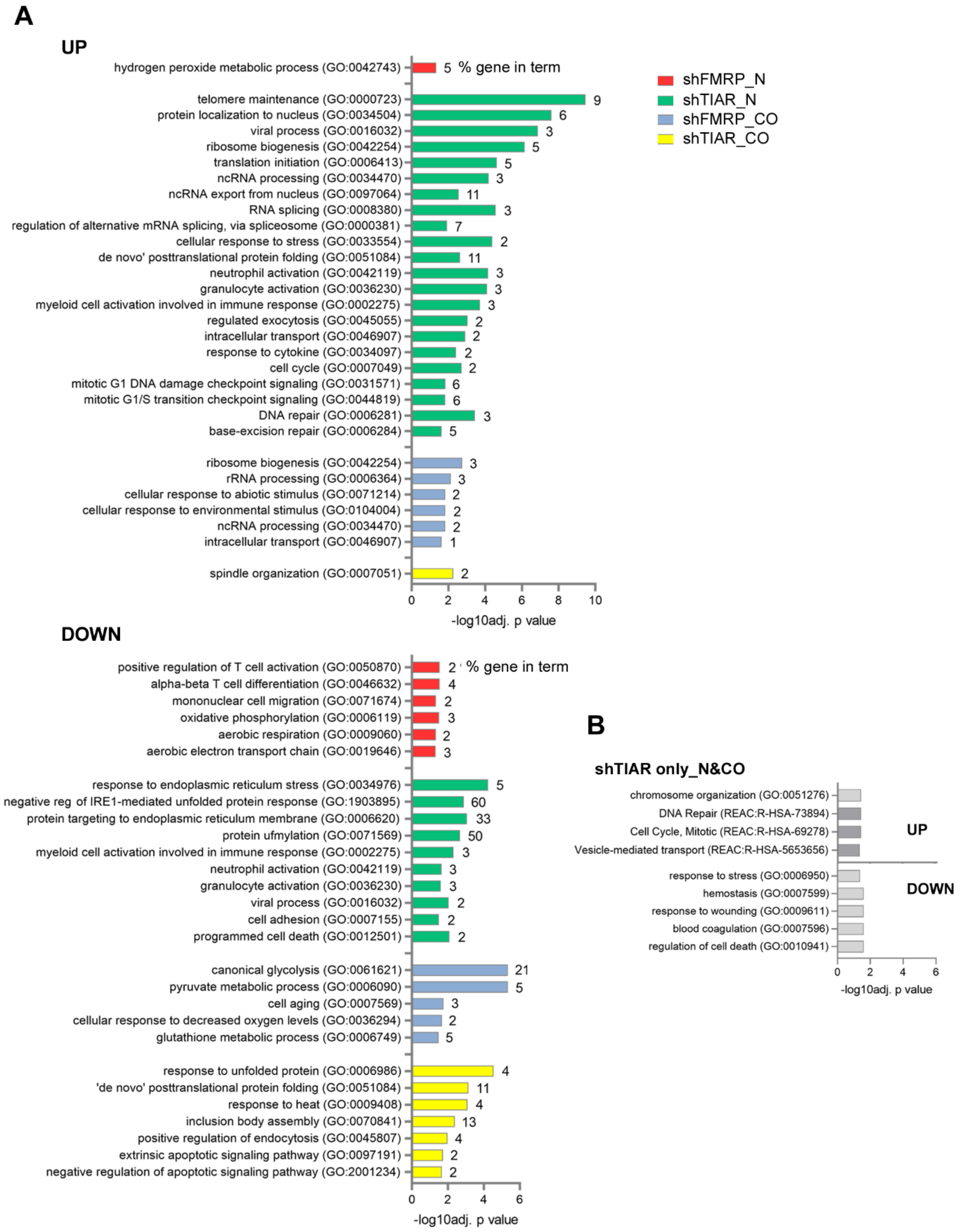

Figure S8. Modification of the nascent proteome by TIAR or FMRP silencing, related to Figure 7. K562 cells transduced with shNEG, shTIAR or shFMRP (shRBP) were cultured for 72h in normoxia mono-culture (N) or hypoxia co-culture (CO). Newly synthetized peptides labeled for 4h with Met analog (L-HPG) and 'heavy' or 'medium' Lys or Arg, after the click chemical reaction with biotin-azide were enriched by binding to beads. Identification and quantification of the peptides performed using SILAC-MS based on the 'heavy' to 'medium' peptides intensity detected, then calculated ratio shRBP to shNEG. Results from 3 independent experiments are presented. **(A)** Comparison of proteins significantly upregulated (fold change log2>0.6, UP) or downregulated (log2<-0.6, DOWN) in shTIAR or shFMRP versus shNEG, in N or CO conditions (color of bars corresponds the legend on the right); Proteins exclusively regulated by shTIAR or shFMRP in respective conditions analyzed for functional enrichment according to annotation in Gene Ontology Biological Processes (GO:BP) using g:GOSt/g:Profiler. The enrichment values of selected terms with a Benjamin-Hochberg (Benj.Hoch.) FDR ≤0.05 are presented. Numbers by the bars indicate % of terms' genes in the indicated GO:BP present in the sample. **(B)** Proteins exclusively regulated by shTIAR in both N and CO.

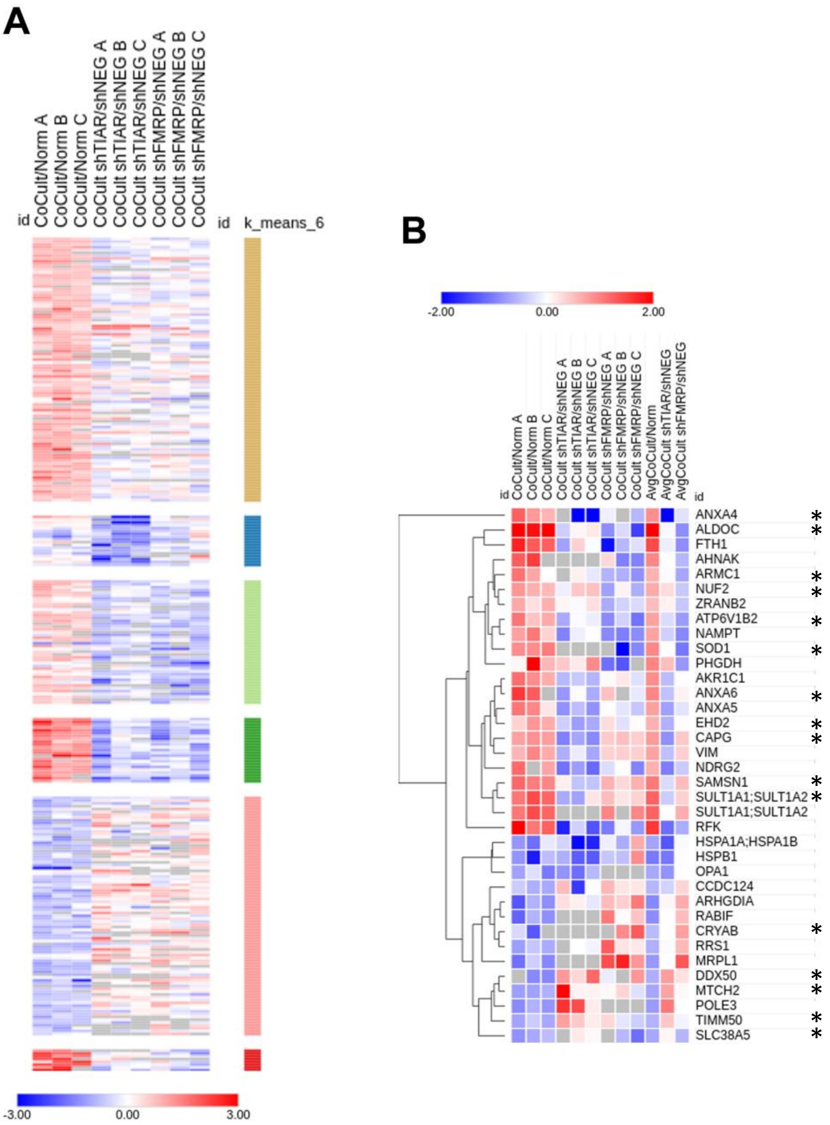

Figure S9. Results of a computational comparison of BONCAT-MS results, related to Figure 7. The heatmaps present log2 fold change of protein level in K562 cells in CO vs N conditions referred to change of these protein level upon shRBP (shTIAR or shTIAR vs shNEG) in CO conditions. **(A)** Non-inputed log2 fold changes of protein expression between the indicated pairs of conditions shown as a heatmap, with the rows (i.e. proteins) clustered using the Euclidean distance and the k-means algorithm, for k=6. **(B)** Subset of the data shown in A, for the proteins that *both* markedly changed expression between CoCult and Norm (absolute average log2 change > 0.5) *and* responded differently to the silencing of TIAR then to the silencing of FMRP (the absolute difference between the corresponding average log2 fold changes >0.5). The rows (i.e. proteins) were clustered hierarchically using the Euclidean distance; *- proteins with putative IRES element in the corresponding mRNA according to the Human IRES Atlas database. **(A-B)** Results from 3 independent experiments (designated A, B and C following the sample name) are presented. Analysis limited to proteins identified in at least 2 comparisons (CO/N and either of the shRBP/shNEG). Log2 fold changes represented using the color scales shown, with lack of change in white, log2 change < 0 in blue, log2 change > 0 in red.
